# The uppermost monoterpenes improving *Cinnamomum camphora* thermotolerance by serving signaling functions

**DOI:** 10.3389/fpls.2022.1072931

**Published:** 2022-12-15

**Authors:** Chenyi Xu, Bin Wang, Qingyun Luo, Yuandan Ma, Tiefeng Zheng, Yingying Wang, Yuyan Cai, Zhaojiang Zuo

**Affiliations:** ^1^ State Key Laboratory of Subtropical Silviculture, Zhejiang A&F University, Hangzhou, China; ^2^ Zhejiang Provincial Key Laboratory of Forest Aromatic Plants-based Healthcare Functions, Zhejiang A&F University, Hangzhou, China

**Keywords:** *Cinnamomum camphora*, gene expression, photosynthesis, reactive oxygen species, thermotolerance mechanism, uppermost monoterpene

## Abstract

Terpenes serve important functions in enhancing plant thermotolerance. *Cinnamomum camphora* mainly has eucalyptol (EuL), camphor (CmR), linalool (LnL) and borneol (BeL) chemotypes basing on the uppermost monoterpenes. To reveal the thermotolerance mechanisms of these uppermost monoterpenes (eucalyptol, camphor, linalool, and borneol) in *C. camphora*, we surveyed the ROS metabolism and photosynthesis in the 4 chemotypes fumigated with the corresponding uppermost monoterpene after fosmidomycin (Fos) inhibiting monoterpene synthesis under high temperature at 38°C (Fos+38°C+monoterpene), and investigated the related gene expression in EuL and CmR. Meanwhile, the thermotolerance differences among the 4 uppermost monoterpenes were analyzed. In contrast to normal temperature (28°C), ROS levels and antioxidant enzyme activities in the 4 chemotypes increased under 38°C, and further increased in the treatment with Fos inhibiting monoterpene synthesis at 38°C (Fos+38°C), which may be caused by the alterations in expression of the genes related with non-enzymatic and enzymatic antioxidant formation according to the analyses in EuL and CmR. Compared with Fos+38°C treatment, Fos+38°C+monoterpene treatments lowered ROS levels and antioxidant enzyme activities for the increased non-enzymatic antioxidant gene expression and decreased enzymatic antioxidant gene expression, respectively. High temperature at 38°C reduced the chlorophyll and carotenoid content as well as photosynthetic abilities, which may result from the declined expression of the genes associated with photosynthetic pigment biosynthesis, light reaction, and carbon fixation. Fos+38°C treatment aggravated the reduction. In contrast to Fos+38°C treatment, Fos+38°C+monoterpene treatments increased photosynthetic pigment content and improved photosynthetic abilities by up-regulating related gene expression. Among the 4 uppermost monoterpenes, camphor showed strong abilities in lowering ROS and maintaining photosynthesis, while eucalyptol showed weak abilities. This was consistent with the recovery effects of the gene expression in the treatments with camphor and eucalyptol fumigation. Therefore, the uppermost monoterpenes can enhance *C. camphora* thermotolerance as signaling molecules, and may have differences in the signaling functions.

## 1 Introduction

Terpenes are one of the major group of volatile organic compounds (VOCs) released from plants (Holopainen and Blande, 2013), of which isoprene, monoterpenes and diterpenes are formed *via* methylerythritol-4-phosphate pathway (MEP) in plastids, and sesquiterpenes are formed *via* mevalonate pathway (MVA) in cytoplasm (Zuo, 2019). The formation and emission of terpenes are regulated by environmental conditions, such as temperature, CO_2_, water, light, insect feeding (Zuo et al., 2019; Zheng et al., 2020; Tian et al., 2021). For the emitters, terpenes contribute to floral scents, fruit aromas and crop quality, and play important roles in attracting pollinators and seed dispersers (Mostafa et al., 2022). In ecosystems, terpenes not only serve important functions in ecological relationships between emitters and other plants or insects (Llusia et al., 2013; Vivaldo et al., 2017), but also play essential roles in protecting emitters against various stresses (Holopainen, 2011; Zuo, 2019).

Climate warming is one of global challenges not only for humans but also for plants, as high temperature seriously impacts plants by inducing reactive oxygen species (ROS) accumulation and damaging photosystems (Mathur et al., 2014; Wang et al., 2014). It is widely reported that high temperature can promote terpene emission from plants (Jardine et al., 2017; Guidolotti et al., 2019). Meanwhile, the terpene emission is beneficial to plant thermotolerance (Holopainen and Blande, 2013; Zuo et al., 2017; Tian et al., 2020). Isoprene-emitting *Arabidopsis thaliana* transformed with isoprene synthase gene (*ISPS*) enhanced the tolerance to high temperature at 40°C and even 60°C (Loivamäki et al., 2007; Sasaki et al., 2007; Zuo et al., 2019). After knocking down the *ISPS*, grey poplar (*Populus pruinosa* Schrenk) decreased photosystem II (PSII) efficiency and CO_2_ assimilation rate in exposure to high temperature (Behnke et al., 2007). When isoprene emission from *Vismia guianensis* was inhibited by fosmidomycin (Fos) blocking MEP pathway, the plant reduced PSII efficiency under high temperature (Rodrigues et al., 2020). In fumigation with monoterpenes, *Quercus ilex* (Loreto et al., 1998; Peñuelas and Llusià, 2002) and *Q. suber* (Delfine et al., 2000) improved thermotolerance by declining leaf damage and maintaining photosynthetic abilities. In addition, monoterpenes and isoprene can also enhance plant tolerance to O_3_, as blocking their synthesis by Fos resulted in ROS accumulation and photosynthesis reduction under O_3_ stress (Loreto and Velikova, 2001; Loreto et al., 2004).

For the thermotolerance mechanisms of isoprene and monoterpenes, they has been mainly hypothesized that these small molecules can stabilize chloroplast membranes by intercalating into membranes against leakiness, and directly quench ROS as antioxidants (Sharkey et al., 2008; Velikova et al., 2011; Zuo et al., 2017). However, isoprene cannot dissolve into cellular membranes in great quantity, and is not involved in the formation of thylakoid membrane acyl lipids (Harvey et al., 2015). Meanwhile, there is a lack of an *in vivo* evidence about isoprene and monoterpenes with very low concentration quenching ROS in plant cells. Thus, the two hypotheses about the thermotolerance mechanisms are suspected by more and more people.

Recently, a new role about isoprene and monoterpenes regulating gene expression has been reported. In myrcene or ocimene fumigation, *A. thaliana* up-regulated the expression of the genes as transcription factors or involving in stress or defense responses (Godard et al., 2008). Riedlmeier et al. (2017) and Wenig et al. (2019) found that α-pinene and β-pinene fumigation can raise expression of the genes related with salicylic acid-mediated innate immune responses. In exposure to isoprene, *A. thaliana* changed expression of the genes that coded for proteins functionally associated with flavonoid and phenylpropanoid biosynthesis, cell wall synthesis, photosynthetic light reaction, stress responses, etc. (Harvey and Sharkey, 2016). When *A. thaliana* and tobacco (*Nicotiana tabacum*) were transferred into *ISPS* to become isoprene emitters, altered expression was found in the genes related with signaling networks and growth regulators, and up-regulated expression was found in the genes related with stress tolerance (Zuo et al., 2019). These findings demonstrate that monoterpenes and isoprene should play important signaling roles in plant tolerating stresses (Lantz et al., 2019; Zuo et al., 2019; Tian et al., 2020).

*Cinnamomum camphora* (L.) J. Presl releases a wide spectrum of terpenes, and is used as Chinese herbal medicine, a flavor and fragrance agent, and excellent evergreen tree species for landscaping, etc. (Shi et al., 2016; Li et al., 2018; Yakefu et al., 2018). This species has 4 main chemotypes, including eucalyptol (EuL), camphor (CmR), linalool (LnL) and borneol (BeL) chemotypes (Luo et al., 2021). During one year, the 4 chemotypes of *C. camphora* improved monoterpene emission in hot months by raising expression of the genes associated with monoterpene synthesis (Tian et al., 2021). *C. camphora* is widely cultivated in southern China, an area frequently harmed by high temperature (Ma et al., 2019). In outdoor experiments, ROS accumulation and photosynthetic ability decline were found in the 4 chemotypes of adult *C. camphora* with inhibiting monoterpene synthesis under high temperature weather (Xu et al., 2022). Similar results were also found in indoor seedlings of EuL under high temperature, suggesting that monoterpene emission is beneficial to the plant tolerating high temperature (Zuo et al., 2017). When monoterpene synthesis in adult CmR was blocked by Fos, the fumigation with terpinene and β-pinene enhanced the plant thermotolerance by altering expression of 73 genes, demonstrating that the 2 monoterpenes may act as signals to enhance the plant thermotolerance (Tian et al., 2020).

Eucalyptol, camphor, linalool and borneol are the uppermost monoterpenes of *C. camphora*. Their emission amount more than 50% of total monoterpene emission amount in the corresponding chemotype, and seriously declined by more than 91% when Fos blocked the monoterpene synthesis (Tian et al., 2021). However, it is still unknown the roles of these uppermost monoterpenes in *C. camphora* tolerating high temperature. Therefore, in the present study, the ROS metabolism and photosynthetic abilities were investigated in the 4 main chemotypes of *C. camphora* that were fumigated with the corresponding uppermost monoterpenes under high temperature after Fos blocking monoterpene synthesis. The related gene expression was analyzed in EuL and CmR. Meanwhile, the thermotolerance differences among the uppermost monoterpenes were analyzed. The present findings not only uncover the thermotolerance mechanisms of the uppermost monoterpenes in *C. camphora*, but also provide a solid evidence for the new insight that monoterpenes and isoprene serve signaling functions in plants tolerating high temperature.

## 2 Materials and methods

### 2.1 Monoterpene fumigation

In each chemotype (EuL, CmR, LnL, and BeL), a random selection was carried out to obtain 4 adult *C. camphora* plants, whose growth conditions were detailedly described by Tian et al. (2021). For each selected plant, its southern branches with 15–18 leaves were randomly selected for the monoterpene fumigation. These branches were cut at 4: 00 PM, and immediately put into Hogland nutrient solution. In each chemotype, the branches from 4 plants were performed random grouping, and they were divided into 5 groups, with each branch from each plant as a replicate.

These branches were placed into a growth chamber with light intensity at 300 μmol·m^-2^·s^-1^ and temperature at 28°C for adaption. After 2 h, the branches in groups 3-5 were sprayed with 30 μM Fos to block monoterpene synthesis (Tian et al., 2020), while the branches in groups 1 and 2 were sprayed with distilled water. After that, these branches were still kept in the growth chamber for 4-h light and 8-h dark, and put into airtight transparent glass boxes (L×W×H, 35×24×19 cm), with each group in a box. For the groups 4 and 5, a piece of watch glass (diameter of 10 cm) was put into each box, and a certain volume of the uppermost monoterpene solution (consistent with the chemotype) was sprayed onto it. After full volatilization (about 1 h for the highest concentration), the monoterpene concentration in the box was 1 μM and 5 μM, respectively. Then, the group 1 (28°C) was till kept in the growth chamber at 28°C, while the group 2 (38°C), group 3 (Fos+38°C), group 4 (Fos+38°C+monoterpene 1) and group 5 (Fos+38°C+monoterpene 5) were placed into another growth chamber for the treatment with high temperature at 38°C. At the 5^th^ h during treatment, these groups were immediately changed to new preheating boxes (28°C for group 1, and 38°C for other groups) for ventilation, and then added into the uppermost monoterpene solution to perform the same treatment (about 25 min for the full volatilization of 5 µM monoterpene at 38°C) (Detailed procedure in **Supplementary Table 1**). After 10-h fumigation, the 3^rd^ and 4^th^ leaves in each branch from the top were used to measure the ROS levels, thiobarbituric acid reactive substance (TBARS) content, antioxidant enzyme activities, photosynthetic pigment levels and photosynthetic abilities in the 4 chemotypes, as well as the related gene expression in EuL and CmR.

### 2.2 Determination of ROS content

*C. camphora* leaves (0.2 g) were ground to homogenate with phosphate buffer solution (PBS) of 50 mM at pH 7.2 by using liquid nitrogen. After centrifugation at 12000 g at 4°C, the supernatant was collected to estimate O_2_–· content through hydroxylamine oxidation in description of Zuo et al. (2017).

H_2_O_2_ content was estimated following the method described by Tian et al. (2020). The leaf samples (0.5 g) were homogenized with cold acetone, and centrifuged at 12000 g at 4°C. After centrifugation, the extracted solution was mixed with 2 mL ddH_2_O, and extracted with the solution that contained 1 volume of CHCl_3_ and 3 volume of CCl_4_. Then, the upper solution was used to estimate H_2_O_2_ levels by oxidizing xylenol orange.

### 2.3 Assessment of SOD and POD activities

The extraction of superoxide dismutase (SOD) and peroxidase (POD) followed the method of O_2_–· extraction. For their activities, the inhibition of p-nitro blue tetrazolium chloride reduction was used to estimate the SOD activity, with the maximum absorption at 560 nm, while the oxidation of guaiacol was used to estimate the POD activity, with the maximum absorption at 470 nm (Zuo et al., 2017).

### 2.4 Determination of TBARS content

TBARS was extracted according to the O_2_–· extraction method. The extracts of 1 ml were added into 2 ml 20% trichloroacetic acid which contained 0.5% thiobarbituric acid. The absorbance of the mixture was recorded at 450, 532 and 600 nm, and used to calculate TBARS content by using the formula described by Ma et al. (2019).

### 2.5 Determination of photosynthetic pigment levels

With aiding of a puncher (Φ 6.78 mm), 2 leaf discs were obtained from the 3^rd^ and 4^th^ leaves of the treated branches from the top, and were homogenized with 3 ml 80% acetone. After centrifugation at 8000 g, the supernatant was collected to measure the levels of chlorophyll (Chl) a, Chl b and carotenoids (Car) (Lichtenthaler and Welburn, 1983).

### 2.6 Assessment of Chl fluorescence transients

Following the previous procedure (Zuo et al., 2017), *C. camphora* leaves were dark-adapted for 30 min, and measured with YZQ500 Chl-fluorescence analyzer (YZQ Technology Co., China) for the Chl fluorescence transients (OJIP). To analyze the OJIP curve, the PSII maximum quantum yield of primary photochemistry (φPo) and non-photochemical deexcitation (φD_O_) were calculated following the presentation of Strasser et al. (2004).

### 2.7 Transcriptome analysis

In EuL and CmR, 3 branches were randomly selected in each treatment. Total RNA were extracted from the 3^rd^ and 4^th^ leaves from the branch with an extraction kit. They were reverse transcribed into cDNA with reverse transcriptase (RNaseH) and random hexamer primer, according to the method described by Tian et al. (2020). After amplification through polymerase chain reaction (PCR), the cDNA from each sample (branch) was constructed a library for the sequence analysis in Novogene Bioinformatics Technology Co. (Beijing, China). (Zuo et al., 2018)

*C. camphora* transcriptome was assembled using the clean reads with Trinity software (Grabherr et al., 2011). The expression levels of the genes were determined according to the RNA-Seq method (Li and Dewey, 2011). DESeq R package and KOBAS were used to perform the analysis of differential expression genes with *P*< 0.05 and fold change >1 and annotation, respectively (Mao et al., 2005; Anders and Huber, 2012).

### 2.8 Statistical analysis

The differences among the treatments were analyzed with one-way ANOVA following the Tukey test.

## 3 Results

### 3.1 Effects of the uppermost monoterpenes on ROS content in *C. camphora*

**Figure 1:**
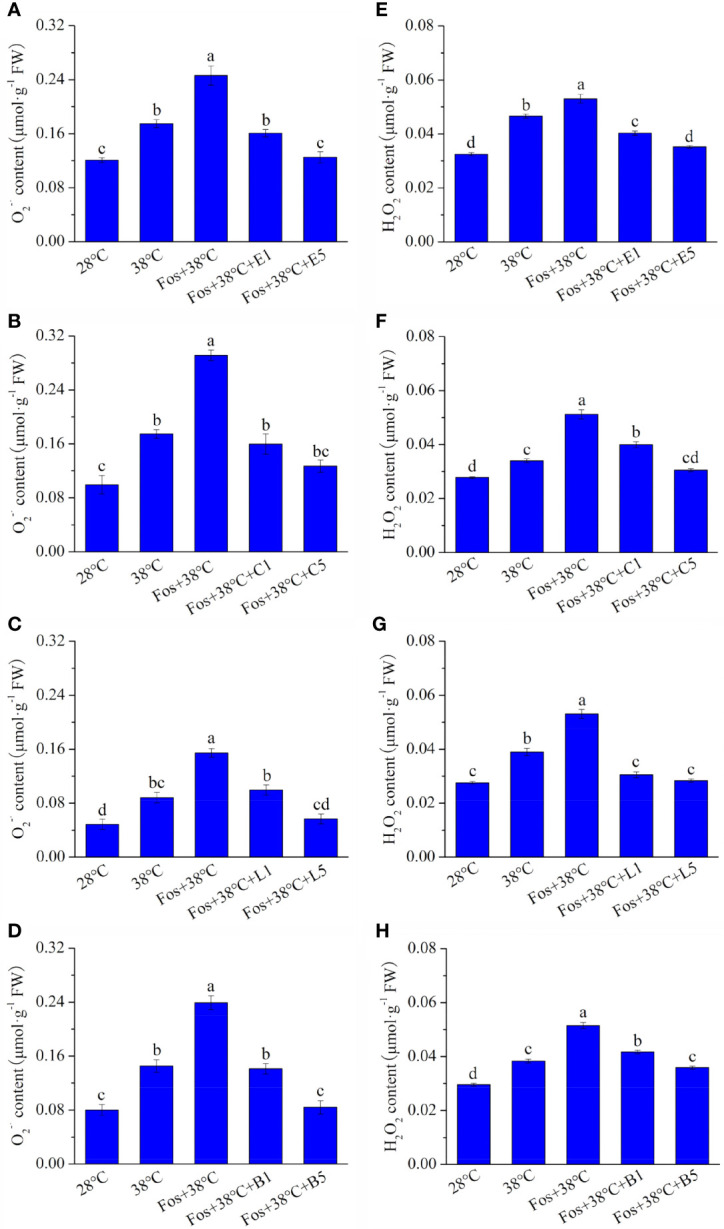
Effects of the uppermost monoterpenes on O_2_–· **(A–D)** and H_2_O_2_ **(E–H)** levels in *C. camphora*. **(A, E)** Eucalyptol chemotype (EuL); **(B, F)** Camphor chemotype (CmR); **(C, G)** Linalool chemotype (LnL); **(D, F)** Borneol chemotype (BeL). 28°C, 38°C, and Fos+38°C: *C. camphora* was treated with normal temperature, high temperature, and high temperature with fosmidomycin (Fos) pretreatment, respectively. Fos+38°C+E1 and Fos+38°C+E5: EuL blocked monoterpene synthesis with Fos was fumigated with 1 and 5 μM eucalyptol at 38°C, respectively. Fos+38°C+C1 and Fos+38°C+C5: CmR blocked monoterpene synthesis with Fos was fumigated with 1 and 5 μM camphor at 38°C, respectively. Fos+38°C+L1 and Fos+38°C+L5: LnL blocked monoterpene synthesis with Fos was fumigated with 1 and 5 μM linalool at 38°C, respectively. Fos+38°C+B1 and Fos+38°C+B5: BeL blocked monoterpene synthesis with Fos was fumigated with 1 and 5 μM borneol at 38°C, respectively. Different lowercase letters indicate the significant difference at *P*< 0.05. Means ± SE (n = 4).

With respect to 28°C, the O_2_–· content in EuL significantly raised by 44.7% (*P*< 0.05) and 1.04 folds (*P*< 0.05) in the treatments with 38°C and Fos+38°C, respectively. Compared with Fos+38°C treatment, the O_2_–· content significantly decreased by 34.8% (*P*< 0.05) and 49.3% (*P*< 0.05) in the treatments with Fos+38°C+E1 (1 μM eucalyptol) and Fos+38°C+E5 (5 μM eucalyptol), respectively (**Figure 1A**). Similarly, the O_2_–· content in CmR, LnL and BeL also raised to the maximum level in the treatment with Fos+38°C, and remarkably reduced in the treatments with Fos+38°C+camphor, Fos+38°C+linalool and Fos+38°C+borneol, respectively. When the concentration of the 3 monoterpenes was at 5 μM, the O_2_–· content also reduced to the level at 28°C (**Figures 1B–D**).

Similar to O_2_–· content, the fumigation with the 4 uppermost monoterpenes also significantly (*P*< 0.05) declined the H_2_O_2_ content in the corresponding chemotypes, with the decrease gradually enhancing with raising the monoterpene concentration (**Figures 1E–H**).

### 3.2 Effects of the uppermost monoterpenes on membrane damage in *C. camphora*

**Figure 2:**
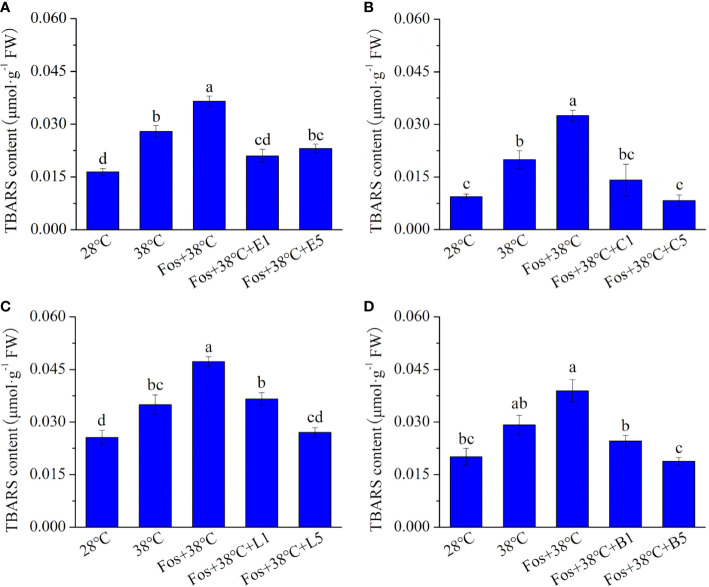
Effects of the uppermost monoterpenes on thiobarbituric acid reactive substance (TBARS) content in *C*. *camphora*. **(A)** Eucalyptol chemotype (EuL); **(B)** Camphor chemotype (CmR); **(C)** Linalool chemotype (LnL); **(D)** Borneol chemotype (BeL). 28°C, 38°C, and Fos+38°C: *C*. *camphora* was treated with normal temperature, high temperature, and high temperature with fosmidomycin (Fos) pretreatment, respectively. Fos+38°C+E1 and Fos+38°C+E5: EuL blocked monoterpene synthesis with Fos was fumigated with 1 and 5 μM eucalyptol at 38°C, respectively. Fos+38°C+C1 and Fos+38°C+C5: CmR blocked monoterpene synthesis with Fos was fumigated with 1 and 5 μM camphor at 38°C, respectively. Fos+38°C+L1 and Fos+38°C+L5: LnL blocked monoterpene synthesis with Fos was fumigated with 1 and 5 μM linalool at 38°C, respectively. Fos+38°C+B1 and Fos+38°C+B5: BeL blocked monoterpene synthesis with Fos was fumigated with 1 and 5 μM borneol at 38°C, respectively. Different lowercase letters indicate the significant difference at *P*< 0.05. Means ± SE (n = 4).

Compared with 28°C, the TBARS content in the 4 chemotypes significantly increased in 38°C treatment, and further increased to the maximum level in the treatment with Fos+38°C. However, remarkable decreases were detected in Fos+38°C+monoterpene treatments compared with Fos+38°C treatment (**Figure 2**).

### 3.3 Effects of the uppermost monoterpenes on antioxidant enzyme activities in *C. camphora*

**Figure 3:**
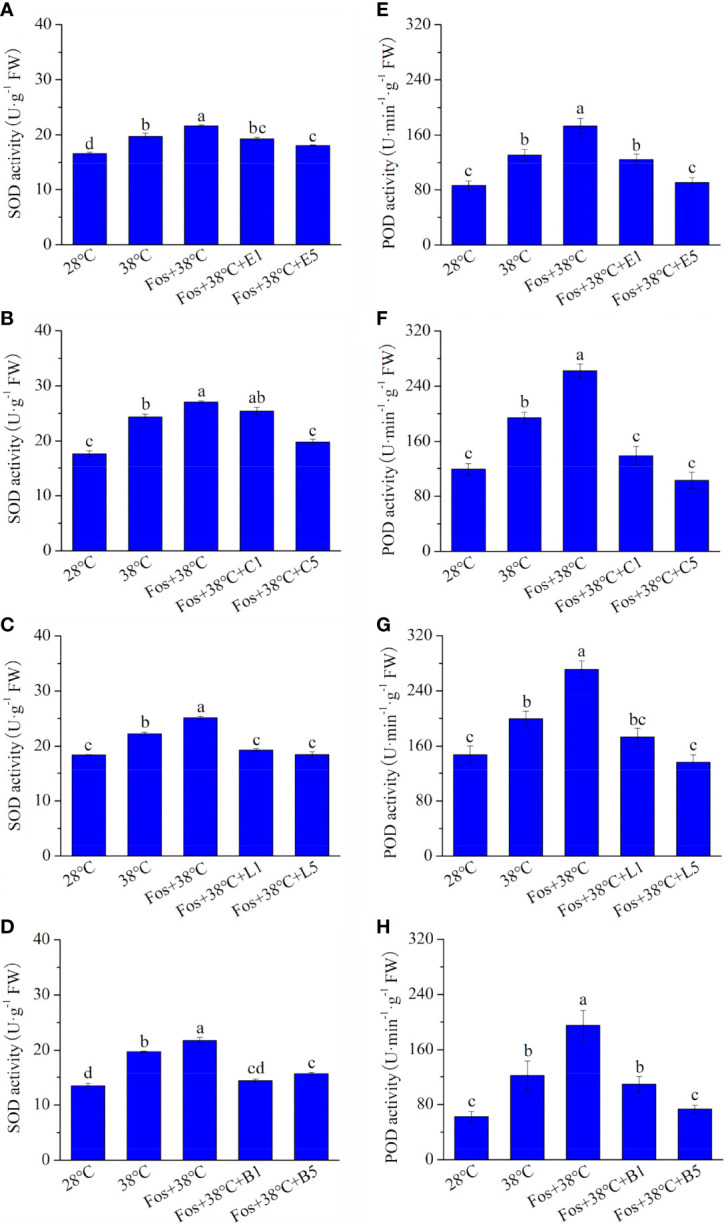
Effects of the uppermost monoterpenes on superoxide dismutase (SOD) **(A–D)** and peroxidase (POD) **(E–H)** activities in *C. camphora*. **(A, E)** Eucalyptol chemotype (EuL); **(B, F)** Camphor chemotype (CmR); **(C, G)** Linalool chemotype (LnL); **(D, F)** Borneol chemotype (BeL). 28°C, 38°C, and Fos+38°C: *C. camphora* was treated with normal temperature, high temperature, and high temperature with fosmidomycin (Fos) pretreatment, respectively. Fos+38°C+E1 and Fos+38°C+E5: EuL blocked monoterpene synthesis with Fos was fumigated with 1 and 5 μM eucalyptol at 38°C, respectively. Fos+38°C+C1 and Fos+38°C+C5: CmR blocked monoterpene synthesis with Fos was fumigated with 1 and 5 μM camphor at 38°C, respectively. Fos+38°C+L1 and Fos+38°C+L5: LnL blocked monoterpene synthesis with Fos was fumigated with 1 and 5 μM linalool at 38°C, respectively. Fos+38°C+B1 and Fos+38°C+B5: BeL blocked monoterpene synthesis with Fos was fumigated with 1 and 5 μM borneol at 38°C, respectively. Different lowercase letters indicate the significant difference at *P*< 0.05. Means ± SE (n = 4).

In the treatments with 38°C and Fos+38°C, the SOD activity in EuL significantly increased by 18.9% (*P*< 0.05) and 30.4% (*P*< 0.05), respectively, in contrast to that at 28°C. Compared with Fos+38°C treatment, the SOD activity significantly decreased by 10.8% (*P*< 0.05) and 16.4% (*P*< 0.05) in Fos+38°C+E1 and Fos+38°C+E5 treatments, respectively (**Figure 3A**). Similarly, the decline was also found in CmR treated with Fos+38°C+camphor, LnL treated with Fos+38°C+linalool, and BeL treated with Fos+38°C+borneol (**Figures 3B–D**).

The POD activity in EuL, CmR, LnL and BeL also raised to the maximum level in the treatment with Fos+38°C, and then reduced in the fumigation with eucalyptol, camphor, linalool and borneol, respectively (**Figures 3E–H**).

### 3.4 Effects of the uppermost monoterpenes on photosynthetic pigment levels in *C. camphora*

The Chl a content in EuL treated with 38°C and Fos+38°C showed remarkable reduction with respect to that at 28°C, and the lowest content was found in the treatment with Fos+38°C. However, a significant (*P*< 0.05) increase was found in Fos+38°C+eucalyptol treatment in contrast to that in Fos+38°C treatment. When the eucalyptol concentration was at 5 μM, the Chl a content was even higher than that at 38°C. For the levels of Chl b and Car, they also showed the similar variations (**Table 1**).

**Table 1:** Effects of the uppermost monoterpenes on photosynthetic pigment content in *C. camphora*.

		Chlorophyll a (μg·mm^-2^)	Chlorophyll b (μg·mm^-2^)	Carotenoids (μg·mm^-2^)
EuL	28°C	1.01 ± 0.01a	0.37 ± 0.01a	0.28 ± 0.01a
	38°C	0.66 ± 0.02c	0.27 ± 0.01c	0.20 ± 0.02bc
	Fos+38°C	0.49 ± 0.02d	0.23 ± 0.01d	0.13 ± 0.01d
	Fos+38°C+E1	0.68 ± 0.01c	0.26 ± 0.02cd	0.19 ± 0.01c
	Fos+38°C+E5	0.87 ± 0.01b	0.33 ± 0.01b	0.24 ± 0.01b
CmR	28°C	0.92 ± 0.03a	0.38 ± 0.01a	0.27 ± 0.01a
	38°C	0.64 ± 0.01c	0.29 ± 0.01b	0.20 ± 0.01c
	Fos+38°C	0.47 ± 0.01d	0.22 ± 0.01c	0.10 ± 0.01d
	Fos+38°C+C1	0.71 ± 0.01b	0.31 ± 0.01b	0.19 ± 0.01c
	Fos+38°C+C5	0.85 ± 0.01a	0.37 ± 0.03a	0.23 ± 0.01b
LnL	28°C	0.80 ± 0.01a	0.30 ± 0.01a	0.23 ± 0.01a
	38°C	0.64 ± 0.01c	0.21 ± 0.03bc	0.17 ± 0.01b
	Fos+38°C	0.56 ± 0.01d	0.16 ± 0.01c	0.12 ± 0.01c
	Fos+38°C+L1	0.71 ± 0.02b	0.24 ± 0.01b	0.16 ± 0.01b
	Fos+38°C+L5	0.79 ± 0.04ab	0.28 ± 0.03ab	0.22 ± 0.02ab
BeL	28°C	0.71 ± 0.02a	0.21 ± 0.01a	0.21 ± 0.01a
	38°C	0.53 ± 0.01d	0.15 ± 0.02b	0.17 ± 0.01b
	Fos+38°C	0.43 ± 0.01c	0.11 ± 0.01c	0.13 ± 0.01c
	Fos+38°C+B1	0.63 ± 0.02b	0.21 ± 0.01a	0.18 ± 0.01ab
	Fos+38°C+B5	0.67 ± 0.01ab	0.21 ± 0.01a	0.18 ± 0.01ab

EuL, Eucalyptol chemotype; CmR, Camphor chemotype; LnL, Linalool chemotype; BeL, Borneol chemotype. 28°C, 38°C, and Fos+38°C: *C. camphora* was treated with normal temperature, high temperature, and high temperature with fosmidomycin (Fos) pretreatment, respectively. Fos+38°C+E1 and Fos+38°C+E5: EuL pretreated with Fos was fumigated with 1 and 5 μM eucalyptol at 38°C, respectively; Fos+38°C+C1 and Fos+38°C+C5: CmR pretreated with Fos was fumigated with 1 and 5 μM camphor at 38°C, respectively; Fos+38°C+L1 and Fos+38°C+L5: LnL pretreated with Fos was fumigated with 1 and 5 μM linalool at 38°C, respectively; Fos+38°C+B1 and Fos+38°C+B5: BeL pretreated with Fos was fumigated with 1 and 5 μM borneol at 38°C, respectively. Different lowercase letters indicate the significant difference at P< 0.05. Means ± SE (n = 4).

In other 3 chemotypes, the photosynthetic pigment content also significantly (*P*< 0.05) declined in 38°C treatment with respect to that at 28°C, and declined to the lowest level in Fos+38°C treatment. In contrast to Fos+38°C treatment, the increase was detected in CmR, LnL and BeL treated with Fos+38°C+camphor, Fos+38°C+linalool and Fos+38°C+borneol, respectively (**Table 1**).

### 3.5 Effects of the uppermost monoterpenes on photosynthetic abilities in *C. camphora*

**Figure 4:**
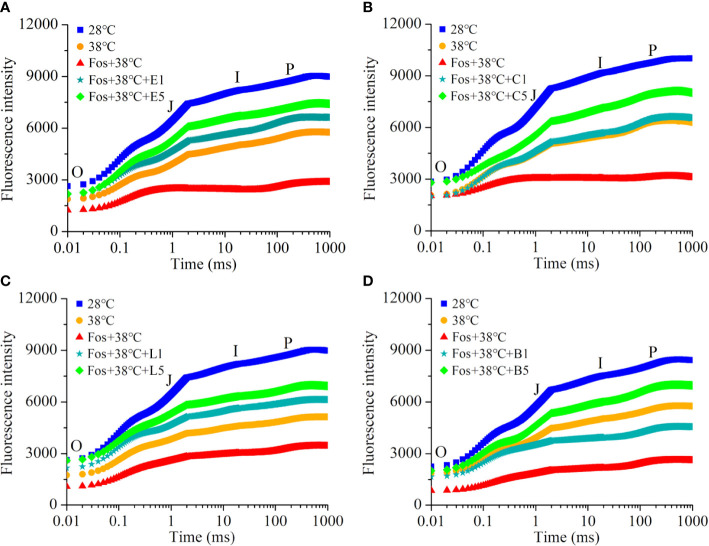
Effects of the uppermost monoterpenes on chlorophyll fluorescence kinetics in *C*. *camphora*. **(A)** Eucalyptol chemotype (EuL); **(B)** Camphor chemotype (CmR); **(C)** Linalool chemotype (LnL); **(D)** Borneol chemotype (BeL). 28°C, 38°C, and Fos+38°C: *C*. *camphora* was treated with normal temperature, high temperature, and high temperature with fosmidomycin (Fos) pretreatment, respectively. Fos+38°C+E1 and Fos+38°C+E5: EuL blocked monoterpene synthesis with Fos was fumigated with 1 and 5 μM eucalyptol at 38°C, respectively. Fos+38°C+C1 and Fos+38°C+C5: CmR blocked monoterpene synthesis with Fos was fumigated with 1 and 5 μM camphor at 38°C, respectively. Fos+38°C+L1 and Fos+38°C+L5: LnL blocked monoterpene synthesis with Fos was fumigated with 1 and 5 μM linalool at 38°C, respectively. Fos+38°C+B1 and Fos+38°C+B5: BeL blocked monoterpene synthesis with Fos was fumigated with 1 and 5 μM borneol at 38°C, respectively. Means (n = 4) are shown.

In contrast to 28°C, the Chl fluorescence intensity in the 4 chemotypes from O to P remarkably decreased in 38°C treatment, and further decreased to the lowest level in the treatment with Fos+38°C. However, a remarkable increase was found in EuL, CmR, LnL and Bel in the treatments with Fos+38°C+eucalyptol, Fos+38°C+camphor, Fos+38°C+linalool and Fos+38°C+borneol, respectively, in contrast to Fos+38°C treatment (**Figure 4**).

Compared with 28°C, φP_O_ in EuL significantly reduced by 12.7% (*P*< 0.05) and 22.5% (*P*< 0.05) in 38°C and Fos+38°C treatments, respectively. In Fos+38°C+eucalyptol treatment, the φP_O_ significantly (*P*< 0.05) raised with respect to that in Fos+38°C treatment, and was remarkably (*P*< 0.05) higher than that at 38°C when eucalyptol concentration was at 5 µM. However, φD_O_ showed reverse variations, and Fos+38°C+eucalyptol treatment lowered its value (**Table 2**).

**Table 2:** Effects of the uppermost monoterpenes on maximum quantum yield of primary photochemistry (φP_O_) and non-photochemical deexcitation (φD_O_) in *C. camphora*.

		φP_O_	φD_O_
EuL	28°C	0.71 ± 0.01a	0.29 ± 0.01d
	38°C	0.62 ± 0.01c	0.38 ± 0.01b
	Fos+38°C	0.55 ± 0.01d	0.45 ± 0.01a
	Fos+38°C+E1	0.64 ± 0.01c	0.36 ± 0.01b
	Fos+38°C+E5	0.67 ± 0.01b	0.33 ± 0.01c
CmR	28°C	0.73 ± 0.01a	0.27 ± 0.01d
	38°C	0.60 ± 0.01c	0.40 ± 0.01b
	Fos+38°C	0.43 ± 0.02d	0.57 ± 0.02a
	Fos+38°C+C1	0.62 ± 0.01c	0.38 ± 0.01b
	Fos+38°C+C5	0.66 ± 0.01b	0.34 ± 0.01c
LnL	28°C	0.71 ± 0.01a	0.29 ± 0.01e
	38°C	0.57 ± 0.01d	0.43 ± 0.01b
	Fos+38°C	0.51 ± 0.01e	0.49 ± 0.01a
	Fos+38°C+L1	0.63 ± 0.01c	0.37 ± 0.01c
	Fos+38°C+L5	0.65 ± 0.01b	0.35 ± 0.01d
BeL	28°C	0.69 ± 0.01a	0.31 ± 0.01e
	38°C	0.57 ± 0.01c	0.44 ± 0.01c
	Fos+38°C	0.41 ± 0.01e	0.59 ± 0.01a
	Fos+38°C+B1	0.51 ± 0.01d	0.49 ± 0.01b
	Fos+38°C+B5	0.65 ± 0.01b	0.35 ± 0.01d

EuL, Eucalyptol chemotype; CmR, Camphor chemotype; LnL, Linalool chemotype; BeL, Borneol chemotype. 28°C, 38°C, and Fos+38°C: *C. camphora* was treated with normal temperature, high temperature, and high temperature with fosmidomycin (Fos) pretreatment, respectively. Fos+38°C+E1 and Fos+38°C+E5: EuL pretreated with Fos was fumigated with 1 and 5 μM eucalyptol at 38°C, respectively; Fos+38°C+C1 and Fos+38°C+C5: CmR pretreated with Fos was fumigated with 1 and 5 μM camphor at 38°C, respectively; Fos+38°C+L1 and Fos+38°C+L5: LnL pretreated with Fos was fumigated with 1 and 5 μM linalool at 38°C, respectively; Fos+38°C+B1 and Fos+38°C+B5: BeL pretreated with Fos was fumigated with 1 and 5 μM borneol at 38°C, respectively. Different lowercase letters indicate the significant difference at P< 0.05. Means ± SE (n = 4).

Similarly, the increase was also found in φP_O_ in CmR treated with Fos+38°C+camphor, LnL treated with Fos+38°C+linalool, and BeL treated with Fos+38°C+borneol, while the decrease in φD_O_ (**Table 2**).

### 3.6 Variation ratio of the physiological indexes with the 4 uppermost monoterpene fumigation

To compare the thermotolerance differences of the 4 uppermost monoterpenes, the variation ratio of these physiological indexes was calculated in Fos+38°C+monoterpene (5 μM) treatments compared with Fos+38°C treatment. For O_2_–· content, the high reduction ratio was detected in Fos+38°C+C5, Fos+38°C+L5 and Fos+38°C+B5 treatments, while the low in Fos+38°C+E5 treatment. For H_2_O_2_ content, the high reduction ratio was found in Fos+38°C+C5 and Fos+38°C+L5 treatments, while the low in Fos+38°C+E5 and Fos+38°C+B5 treatments. Although SOD activity and Chl b content exhibited the maximum variation ratio in Fos+38°C+B5 treatment, their variation ratio in Fos+38°C+C5 treatment was significantly (*P*< 0.05) higher than that in Fos+38°C+E5 treatment. In terms of other indexes (except Chl a content), they always showed strong variation in Fos+38°C+C5 treatment, but weak variation in Fos+38°C+E5 treatment. Fos+38°C+C5 treatment caused the maximum increase ratio in Chl a content, but without significant difference with Fos+38°C+E5 treatment (**Table 3**).

**Table 3:** Variation ratio of the physiological indexes in the uppermost monoterpene fumigation (Fos+38°C+monoterpene) compared with non-fumigation (Fos+38°C).

	Fos+38°C+E5	Fos+38°C+C5	Fos+38°C+L5	Fos+38°C+B5
O_2_–· content (%)	-49.4 ± 5.1b	-56.4 ± 3.6ab	-63.3 ± 5.5a	-64.8 ± 4.7a
H_2_O_2_ content (%)	-25.0 ± 3.1b	-40.4 ± 2.1a	-46.6 ± 2.6a	-30.3 ± 1.2b
TBARS content (%)	-36.8 ± 5.5c	-74.6 ± 4.5a	-42.7 ± 3.6c	-51.7 ± 2.5b
SOD activity (%)	-16.6 ± 0.4c	-26.8 ± 1.9b	-26.5 ± 2.0b	-33.9 ± 1.4a
POD activity (%)	-47.6 ± 4.1bc	-51.6 ± 3.4ab	-45.6 ± 4.7c	-62.4 ± 3.0a
Chlorophyll a content (%)	77.6 ± 4.8a	80.6 ± 4.8a	40.4 ± 7.8c	56.0 ± 5.1b
Chlorophyll b content (%)	44.9 ± 3.1c	70.0 ± 8.3b	74.2 ± 5.7b	90.7 ± 6.7a
Carotenoids content (%)	84.9 ± 5.3b	128.0 ± 12.1a	86.0 ± 5.1b	41.4 ± 4.9c
φPo (%)	23.3 ± 1.6c	51.8 ± 5.0a	24.1 ± 3.4c	33.8 ± 2.8b
φD_O_ (%)	-28.0 ± 4.7bc	-39.4 ± 5.8a	-25.9 ± 2.2c	-31.8 ± 1.5ab

Fos+38°C: Treatment with high temperature at 38°C after pretreatment with fosmidomycin (Fos); Fos+38°C+E5: EuL pretreated with Fos was fumigated with 5 μM eucalyptol at 38°C; Fos+38°C+C5: CmR pretreated with Fos was fumigated with 5 μM camphor at 38°C; Fos+38°C+L5: LnL pretreated with Fos was fumigated with 5 μM linalool at 38°C; Fos+38°C+B5: BeL pretreated with Fos was fumigated with 5 μM borneol at 38°C. Positive value indicates the increase, while negative value indicates the decrease. Different lowercase letters indicate the significant difference at *P*< 0.05. Means ± SE (n = 4).

### 3.7 Effects of eucalyptol and camphor on gene expression in *C. camphora*

**Figure 5:**
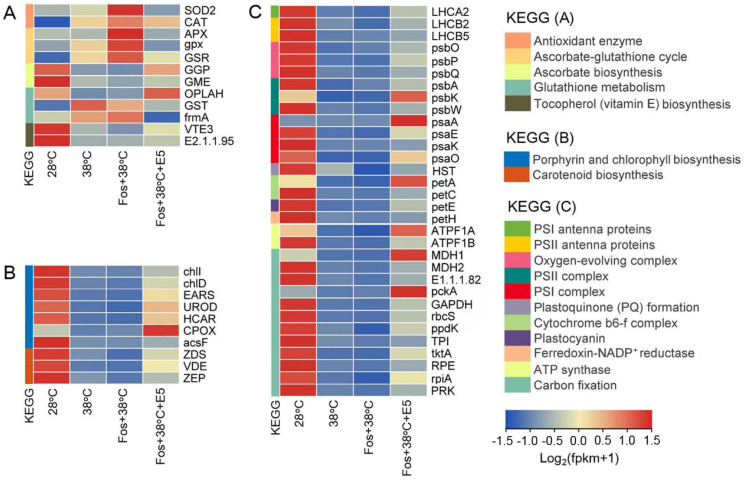
Effects of eucalyptol on gene expression in antioxidation **(A)**, photosynthetic pigment biosynthesis **(B)**, and photosynthetic abilities **(C)** in eucalyptol chemotype of *C*. *camphora* (EuL). 28°C, 38°C, and Fos+38°C: EuL was treated with normal temperature, high temperature, and high temperature with fosmidomycin (Fos) pretreatment, respectively. Fos+38°C+E5: EuL blocked monoterpene synthesis with Fos was fumigated with 5 μM eucalyptol at 38°C. KEGG: Kyoto encyclopedia of genes and genomes pathways. The heatmap was drawn using the FPKM (fragments per kilobase per million mapped reads) by using the software R packages pheatmap 1.0.12. Means (n = 3) are shown.

Compared with 28°C, EuL treated with 38°C changed expression of 12 genes related with antioxidation, including 2 genes encoding antioxidant enzymes (*SOD2* and *CAT*), 3 genes in ascorbate (AsA)-glutathione (GSH) cycle (*APX*, *gpx*, and *GSR*), 2 genes in AsA biosynthesis (*GGP* and *GME*), 3 genes in GSH metabolism (*OPLAH*, *GST*, and *frmA*), and 2 genes in tocopherol (vitamin E, VE) biosynthesis (*VTE3* and *E2.1.1.95*). This alteration further aggravated in the treatment with Fos+38°C. Interestingly, the expression levels of these genes in Fos+38°C+E5 treatment were similar with or trended to that at 28°C. The similar alterations were also found in expression of 7 genes in porphyrin and Chl biosynthesis (*chlI*, *chlD*, *EARS*, *UROD*, *HCAR*, *CPOX*, and *acsF*), 3 genes in carotenoid biosynthesis (*ZDS*, *VDE*, and *ZEP*), 1 gene encoding PSI antenna proteins (*LHCA2*), 2 genes encoding PSII antenna proteins (*LHCB2* and *LHCB5*), 3 genes in oxygen-evolving complex (*psbO*, *psbP*, and *psbQ*), 3 genes in PSII complex (*psbA*, *psbK*, and *psbW*), 4 genes in PSI complex (*psaA*, *psaE*, *psaK*, and *psaO*), 1 gene encoding homogentisate solanesyltransferase for plastoquinone (PQ) formation (*HST*), 2 genes associated with cytochrome b_6_-f complex (Cytb_6_-f) (*petA* and *petC*), 1 gene coding for plastocyanin (*petE*), 1 gene coding for ferredoxin-NADP^+^ reductase (*petH*), 2 genes coding for ATP synthase (*ATPF1A* and *ATPF1B*), and 12 genes in carbon fixation (*MDH1*, *MDH2*, *E1.1.1.82*, *pckA*, *GAPDH*, *rbcS*, *ppdK*, *TPI*, *tktA*, *RPE*, *rpiA*, and *PRK*) (**Figure 5**). The detail functions and expression levels of these genes were provided in **Supplementary Tables 2–4**.

**Figure 6:**
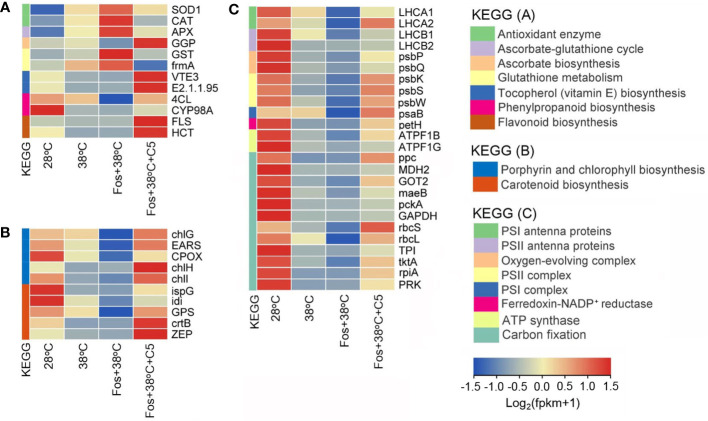
Effects of camphor on gene expression in antioxidation **(A)**, photosynthetic pigment biosynthesis **(B)**, and photosynthetic abilities **(C)** in camphor chemotype of *C*. *camphora* (CmR). 28°C, 38°C, and Fos+38°C: CmR was treated with normal temperature, high temperature, and high temperature with fosmidomycin (Fos) pretreatment, respectively. Fos+38°C+C5: CmR blocked monoterpene synthesis with Fos was fumigated with 5 μM camphor at 38°C. KEGG: Kyoto encyclopedia of genes and genomes pathways. The heatmap was drawn using the FPKM (fragments per kilobase per million mapped reads) by using the software R packages pheatmap 1.0.12. Means (n = 3) are shown.

For antioxidation, CmR treated with 38°C up-regulated expression of 2 genes encoding antioxidant enzymes (*SOD1* and *CAT*), 1 gene in AsA-GSH cycle (*APX*), and 2 genes in GSH metabolism (*GST* and *frmA*), but down-regulated 1 gene in AsA biosynthesis (*GGP*), 2 genes in VE biosynthesis (*VTE3* and *E2.1.1.95*), 2 genes in phenylpropanoid biosynthesis (*4CL* and *CYP98A*), and 2 genes in flavonoid biosynthesis (*FLS* and *HCT*). For photosynthetic pigment biosynthesis, 38°C treatment down-regulated expression of 5 genes in porphyrin and Chl biosynthesis (*chlG*, *EARS*, *CPOX*, *chlH*, and *chlI*), and 5 genes in Car biosynthesis (*ispG*, *idi*, *GPS*, *crtB*, and *ZEP*). For the photosynthetic abilities, 38°C treatment down-regulated expression of 2 genes encoding PSI antenna proteins (*LHCA1* and *LHCA2*), 2 genes encoding PSII antenna proteins (*LHCB1* and *LHCB2*), 2 genes in oxygen-evolving complex (*psbP* and *psbQ*), 3 genes in PSII complex (*psbK*, *psbS*, and *psbW*), 1 gene in PSI complex (*psaB*), 1 gene encoding ferredoxin-NADP^+^ reductase (*petH*), 2 genes encoding ATP synthase (*ATPF1B* and *ATPF1G*), and 12 genes in carbon fixation (*ppc*, *MDH2*, *GOT2*, *maeB*, *pckA*, *GAPDH*, *rbcS*, *rbcL*, *TPI*, *tktA*, *rpiA*, and *PRK*). These alterations were further aggravated in the treatment with Fos+38°C, but their expression in the treatment with Fos+38°C+C5 trended to the levels at 28°C (**Figure 6**; **Supplementary Tables 5–7**).

## 4 Discussion

Fos is an effective inhibitor for isoprene and monoterpene synthesis by blocking MEP pathway (Tian et al., 2021). In exposure to O_3_, ROS accumulation was detected in *P. australis* leaves with blocking isoprene synthesis by Fos and in *Q. ilex* leaves with blocking monoterpene synthesis (Loreto and Velikova, 2001; Loreto et al., 2004). Meanwhile, *P. australis* that was blocked isoprene synthesis accumulated ROS under high temperature stress (Velikova and Loreto, 2005). Under high temperature, EuL seedlings and adult CmR plants remarkably increased ROS and TBARS content after their monoterpene synthesis was blocked (Zuo et al., 2017; Tian et al., 2020). In this study, the similar increase was also found in the 4 chemotypes of *C. camphora* in the treatment with Fos+38°C. Moreover, Fos+38°C+monoterpene (eucalyptol, camphor, linalool, and borneol) treatments significantly declined the ROS and TBARS content, demonstrating that the 4 uppermost monoterpenes served important functions in regulating ROS levels (**Figures 1**, **2**). Although monoterpenes can quench ROS and free radicals *in vitro* (Wojtunik et al., 2014), their scavenging abilities against ROS *in vivo* are suspected for their internal low concentration and lacking a direct scavenging evidence.

ROS accumulation can cause oxidative stress, which is harmful to the cells. There are a large and integrated non-enzymatic and enzymatic antioxidants in plants to regulate ROS levels and reduce oxidative stress. For non-enzymatic antioxidants, Car, VE, AsA, GSH and flavonoids exhibit strong antioxidant abilities (Koopman et al., 2010; Ma et al., 2019; Shen et al., 2022). In the 4 chemotypes, Car content significantly decreased in 38°C treatment and further decreased in Fos+38°C treatment (**Table 1**), which should result from the down-regulation of the genes related with Car biosynthesis, such as *ZDS*, *VDE* and *ZEP* in EuL, as well as *ispG*, *idi*, *GPS*, *crtB* and *ZEP* in CmR. Compared with Fos+38°C treatment, Car content increased in Fos+38°C+monoterpene treatments for the up-regulation of the related genes. Meanwhile, the genes in VE, AsA, GSH and flavonoids biosynthesis and in GSH metabolic transformation were up-regulated and down-regulated, respectively, in Fos+38°C+monoterpene (eucalyptol and camphor) treatments compared with Fos+38°C treatment (**Figures 5**, **6**; **Supplementary Tables 2, 5**), which were also beneficial to non-enzymatic antioxidant formation. These results were consistent with previous studies in CmR fumigated with terpinene and β-pinene (Tian et al., 2020), suggesting that monoterpenes may regulate expression of the genes associated with non-enzymatic antioxidant formation, and then adjust ROS levels.

Enzymatic antioxidants can scavenge ROS through enzymatic reaction, and the enhancement of their activities is induced by ROS accumulation. In the treatment with Fos associating with high temperature, Eul seedlings and adult CmR plants increased the activities of antioxidant enzymes in response to the ROS accumulation (Zuo et al., 2017; Tian et al., 2020). In this study, the activities of SOD and POD also increased in the 4 chemotypes of *C. camphora* in the treatment with Fos+38°C. Moreover, Fos+38°C+monoterpene treatments declined their activities in contrast to Fos+38°C treatment (**Figure 3**). The corresponding alterations were also found in expression of the genes *SOD2* (encoding SOD) and *CAT* (encoding catalase) in EuL treated with Fos+38°C+E5 (**Figure 5A**, **Supplementary Table 2**), and the genes *SOD1* and *CAT* in CmR treated with Fos+38°C+C5 (**Figure 6A**, **Supplementary Table 5**). AsA-GSH cycle includes several enzymes to serve antioxidant function. *APX*, *gpx* and *GSR* encode AsA peroxidase, GSH peroxidase and GSH reductase, respectively (Caverzan et al., 2012; Passaia et al., 2014; Couto et al., 2016). High temperature and acid rain stresses increased their activities in *C. camphora* to quench ROS (Ma et al., 2019). In this study, the expression of the 3 genes in EuL and 1 gene *APX* in CmR increased in 38°C treatment, further increased in Fos+38°C treatment, and then decreased in Fos+38°C+E5 and Fos+38°C+C5 treatments, which may be caused by the variations of the ROS levels (**Figures 5**, **6**; **Supplementary Tables 2, 5**). It can be speculated that monoterpenes reduce ROS levels by promoting non-enzymatic antioxidant formation, and ROS accumulation resulted from blocking monoterpene formation under high temperature stress raises antioxidant enzyme activities by inducing related gene expression.

In previous studies, Chl and Car content significantly reduced in adult CmR plants in Fos+38°C treatment, but increased in Fos+38°C+β-pinene and Fos+38°C+terpinene treatments, due to the alterations of related gene expression (Tian et al., 2020). Similarly, the down-regulation of the genes in Chl and Car biosynthesis in EuL and CmR may lead to the decrease of Chl and Car content, respectively, in the treatments with 38°C and Fos+38°C. In contrast to Fos+38°C treatment, Fos+38°C+E5 and Fos+38°C+C5 treatments increased the photosynthetic pigment content by raising expression of the related genes (**Figures 5**, **6**; **Supplementary Tables 3, 6**).

In the 4 chemotypes, the Chl fluorescence intensity (O to P) decreased in 38°C treatment, and then reduced to the minimum level in the treatment with Fos+38°C (**Figure 4**), which were similar with previous findings in EuL seedlings and adult CmR plants (Zuo et al., 2017; Tian et al., 2020). The decline of O to J was interpreted as the decline of PQ pool (Strasser et al., 1995), as *HST* that coded for homogentisate solanesyltransferase in PQ biosynthesis was down-regulated in EuL (**Figure 5C**; **Supplementary Table 4**). For J to P, its decrease was caused by the blockage of electron transport at PSII donor side (Gao et al., 2016). The treatments with 38°C and Fos+38°C inhibited expression of the genes associated with PSII complex and PSII oxygen-evolving enhancer proteins in EuL and CmR, which may restrain PSII assembly and water photolysis and lead to the decline of electron supply at PSII donor side. Meanwhile, the down-regulation was also found in the genes associated with the assembly of Cytb_6_-f, PSI and ATP synthase, as well as encoding plastocyanin and ferredoxin-NADP^+^ reductase, which may block electron transport, NADP^+^ reduction and ATP formation, and lower assimilatory power (ATP and NADPH) generation. However, these cases reversed to better statuses in Fos+38°C+E5 and Fos+38°C+C5 treatments (**Figures 5**, **6**; **Supplementary Tables 4, 7**).

Compared with 28°C, the fumigation with 1, 3 and 5 µM eucalyptol at 28°C not changed the φPo and φD_O_ in EuL, indicating that monoterpene fumigation no affect the photosynthetic abilities in *C. camphora* under normal temperature (**Supplementary Figure 1**). In contrast to 38°C, a decline was found in φPo in the 4 chemotypes of *C. camphora* treated with Fos+38°C (**Table 2**). This was similar with the reduction of ΔF/Fm′ in *Q. ilex* with blocking monoterpene synthesis under O_3_ stress (Loreto et al., 2004). For φDo, Fos+38°C treatment aggravated its increase. These demonstrated that an aggravated suppression has happened in the quantum yield and electron transport in Fos+38°C treatment, with massive light energy absorbed by photosynthetic pigments consuming as heat (Zhao et al., 2016). The variations of the 2 Chl fluorescence transient parameters were consistent with that in EuL seedlings and adult CmR plants in Fos treatment associating with high temperature stress (Zuo et al., 2017; Tian et al., 2020). Compared with Fos+38°C treatment, Fos+38°C+monoterpene treatments increased φPo in the 4 chemotypes, but decreased φDo (**Table 2**), indicating that the uppermost monoterpene fumigation was beneficial to maintaining PSII efficiency. This was similar with *Q. ilex* maintaining higher ΔF/Fm′ in fumigation with sabinene, α-pinene and cis-β-ocimene (Loreto et al., 1998), and adult CmR plants maintaining higher PSII efficiency in fumigation with terpinene and β-pinene (Tian et al., 2020).

Under high temperature, blocking monoterpene synthesis reduced photosynthetic rate in *Q. ilex* (Loreto et al., 1998), *Q. suber* (Delfine et al., 2000) and EuL seedlings (Zuo et al., 2017) in indoor experiments. Compared with high temperature, blocking monoterpene synthesis under high temperature reduced the stomatal conductance in EuL seedlings, but raised the intercellular CO_2_ concentration, indicating that the photosynthetic rate reduction with blocking monoterpene synthesis under high temperature is non-stoma limitation. Under high temperature weather, the reduction was also detected in the photosynthetic abilities in the 4 chemotypes of outdoor adult *C. camphora* with blocking monoterpene synthesis (Xu et al., 2022). This reduction should not only result from the decline of the photosynthetic pigment content, PSII efficiency and assimilatory power, but also result from the decline of CO_2_ assimilation abilities, due to the down-regulation of related genes in EuL and CmR (**Figures 5**, **6**; **Supplementary Tables 4, 7**). In contrast to Fos+38°C treatment, Fos+38°C+E5 and Fos+38°C+C5 treatments up-regulated expression of the genes associated with CO_2_ assimilation, suggesting that the uppermost monoterpenes were beneficial to maintaining photosynthesis in *C. camphora* under high temperature.

In fumigation with monoterpenes (α-pinene, β-pinene, myrcene, and ocimene), *A. thaliana* up-regulated expression of the genes that were involved in defense (Godard et al., 2008) and innate immune responses (Riedlmeier et al., 2017; Wenig et al., 2019). Both endogenous (Zuo et al., 2019) and exogenous (Harvey and Sharkey, 2016) isoprene exhibited inducing effects on the gene expression in several biological pathways and stress responses. In previous study, terpinene and β-pinene served important functions in adult CmR tolerating high temperature by adjusting expression of the genes associated with ROS metabolism and photosynthetic abilities (Tian et al., 2020). In this study, the uppermost monoterpenes also exhibited similar roles in improving thermotolerance in *C. camphora*, indicating that they also serve signaling functions.

In previous studies, isoprene improved plant photosynthetic abilities by stabilizing thylakoid membranes (Pollastri et al., 2019), which has been hypothesized by intercalating into membranes against leakiness (Sharkey et al., 2008; Velikova et al., 2011). However, isoprene cannot dissolve into cellular membranes in great quantity, and is not involved in the formation of thylakoid membrane acyl lipids (Harvey et al., 2015). When CmR was treated with Fos+38°C+C5, a reduction was found in the cell membrane damage. The levels of membrane lipid molecules showed variation tendencies to the control at 28°C, and the expression of the genes related with these membrane lipid metabolism was also altered accordingly (submitted). This demonstrates that monoterpenes and isoprene might stabilize thylakoid membranes by regulating related gene expression, which is beneficial to maintaining plant photosynthetic abilities under high temperature.

In the previous study, the fumigation with β-pinene and terpinene at 10 µM (Fos+38°C+β-pinene and Fos+38°C+terpinene) recovered the ROS metabolism and photosynthetic pigment levels in CmR to the levels at 28°C (Tian et al., 2020), while the recovery effects were carried out in fumigation with the 4 uppermost monoterpenes at 5 µM, suggesting that the uppermost monoterpenes had stronger thermotolerance abilities. In contrast to Fos+38°C treatment, the fumigation with the 4 uppermost monoterpenes at 5 µM caused different variation ratio in the ROS metabolism, photosynthetic pigment levels and photosynthetic abilities in the corresponding chemotype. Among of them, camphor fumigation (Fos+38°C+C5) showed high variation ratio in most of indexes, while eucalyptol fumigation (Fos+38°C+E5) showed low variation ratio, indicating that camphor might have strong abilities in reducing ROS levels and maintaining photosynthesis under high temperature, and eucalyptol might have weak abilities (**Table 3**). These differences should be caused by the recovery effects of related gene expression, with high in Fos+38°C+C5 treatment but low in Fos+38°C+E5 treatment, which was similar with different monoterpene (α-pinene, β-pinene, myrcene, and ocimene) fumigation inducing different defense responses in gene expression in *A. thaliana* (Godard et al., 2008; Riedlmeier et al., 2017; Wenig et al., 2019). This indicated that monoterpenes might have different signaling effects in improving *C. camphora* thermotolerance.

## Data availability statement

The data presented in the study are deposited in the NCBI SRA database, accession number PRJNA909422.

## Author contributions

ZZ and CX conceived the main idea of the study. CX, BW, and QL performed the experiments and analyzed the data. YM, TZ, YW, and YC took part in the experiments. ZZ wrote and modified the paper. All authors contributed to the article and approved the submitted version.

## Supplementary material

Supplementary Figure 1 Effects of eucalyptol on the maximum quantum yield of primary photochemistry (φPo) and non-photochemical deexcitation (φD_O_) in eucalyptol chemotype of *C. camphora* under normal temperature. Compared with 28°C, the fumigation with 1, 3 and 5 µM eucalyptol (Eul) at 28°C not changed the φPo and φD_O_, indicating that monoterpene fumigation no affect the photosynthetic abilities in *C. camphora* under normal temperature.
