# Erratum: The uppermost monoterpenes improving *Cinnamomum camphora* thermotolerance by serving signaling functions

**DOI:** 10.3389/fpls.2024.1469613

**Published:** 2024-08-07

**Authors:** Frontiers Production Office

**Affiliations:** Frontiers Media SA, Lausanne, Switzerland

**Keywords:** *Cinnamomum camphora*, gene expression, photosynthesis, reactive oxygen species, thermotolerance mechanism, uppermost monoterpene

Due to a production error, the same image for **Figure 5** and **Figure 6** was used. The correct **Figure 5** can be seen below.

**Figure 5:**
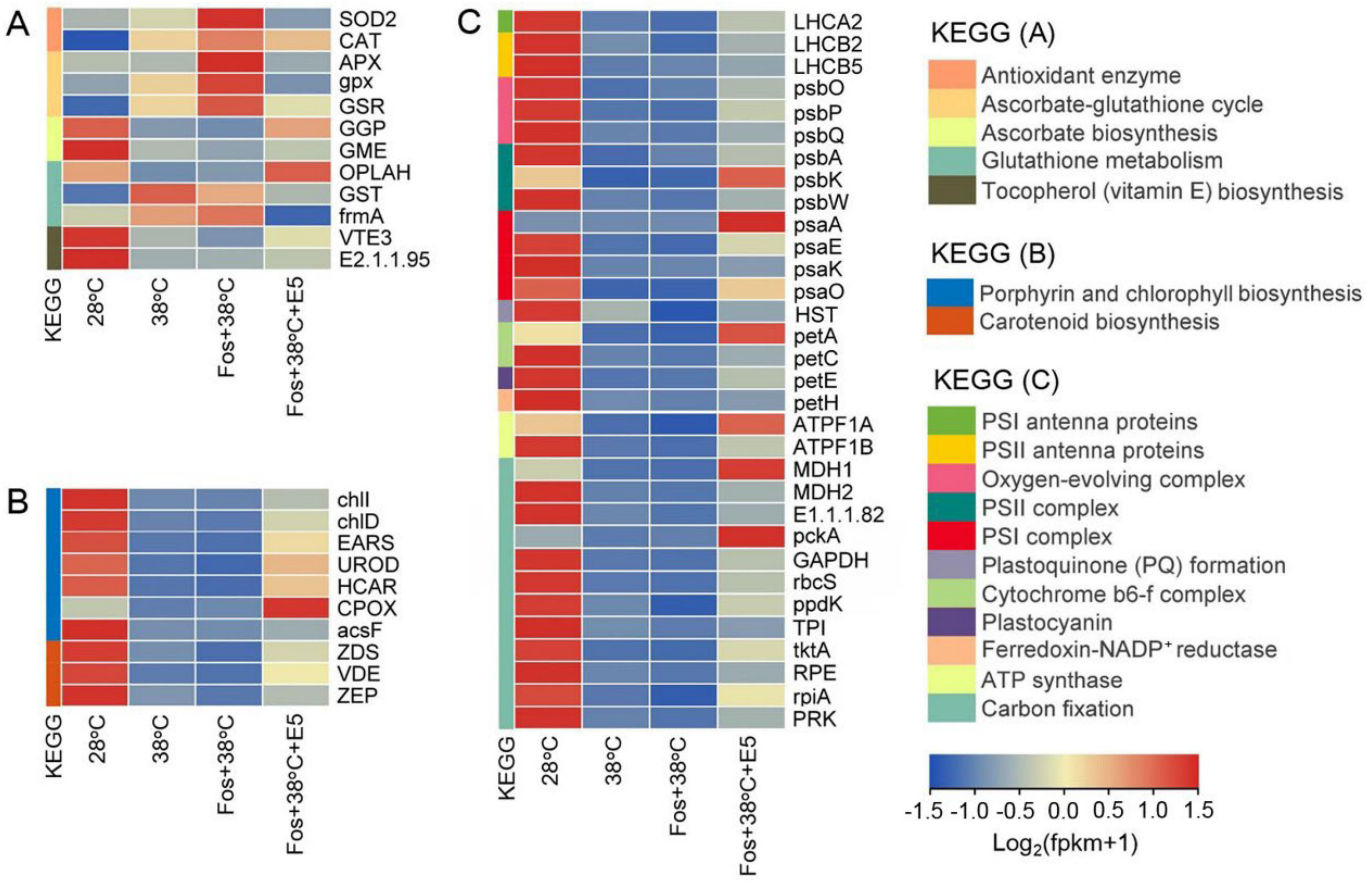
Effects of eucalyptol on gene expression in antioxidation **(A)**, photosynthetic pigment biosynthesis **(B)**, and photosynthetic abilities **(C)** in eucalyptol chemotype of *C*. *camphora* (EuL). 28°C, 38°C, and Fos+38°C: EuL was treated with normal temperature, high temperature, and high temperature with fosmidomycin (Fos) pretreatment, respectively. Fos+38°C+E5: EuL blocked monoterpene synthesis with Fos was fumigated with 5 μM eucalyptol at 38°C. KEGG: Kyoto encyclopedia of genes and genomes pathways. The heatmap was drawn using the FPKM (fragments per kilobase per million mapped reads) by using the software R packages pheatmap 1.0.12. Means (n = 3) are shown.

The publisher apologizes for this mistake.

The original version of this article has been updated.

