# Supplementary figures and images for: The uppermost monoterpenes improving Cinnamomum camphora thermotolerance by serving signaling functions
Source: Front Plant Sci. 2022 Dec 15;13:1072931. doi: 10.3389/fpls.2022.1072931 (PMC9800025; doi:10.3389/fpls.2022.1072931)

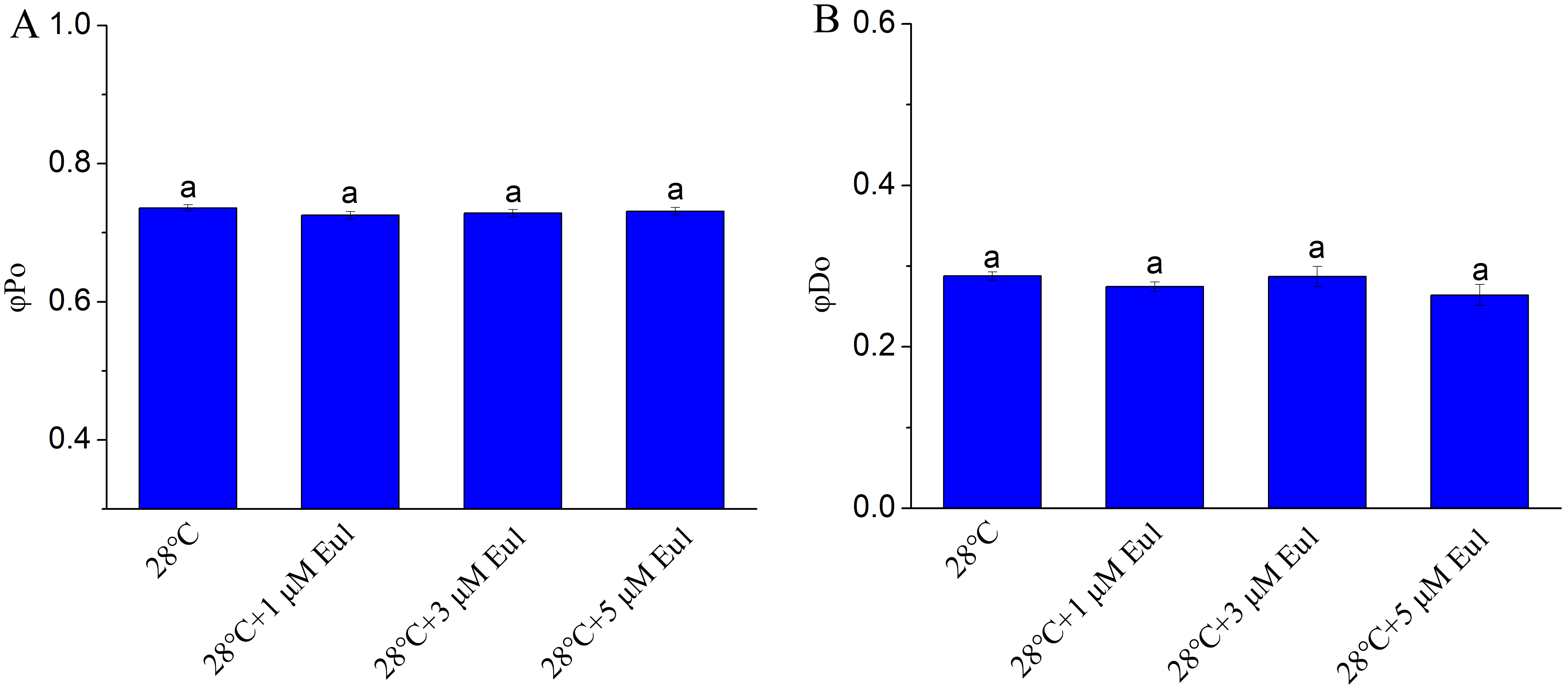

Supplement: Supplementary Figure 1 — Effects of eucalyptol on the maximum quantum yield of primary photochemistry (φPo) and non-photochemical deexcitation (φDO) in eucalyptol chemotype of C. camphora under normal temperature. Compared with 28°C, the fumigation with 1, 3 and 5 µM eucalyptol (Eul) at 28°C not changed the φPo and φDO, indicating that monoterpene fumigation no affect the photosynthetic abilities in C. camphora under normal temperature. [file Image_1.tif]
