# Supplementary material for: The uppermost monoterpenes improving Cinnamomum camphora thermotolerance by serving signaling functions
Source: Front Plant Sci. 2022 Dec 15;13:1072931. doi: 10.3389/fpls.2022.1072931 (PMC9800025; doi:10.3389/fpls.2022.1072931)
Supplement: Supplementary file 2 [file DataSheet_1.doc]

**Supplementary Tables**

| Supplementary Table 1 The procedure of the uppermost monoterpene fumigation | |
| --- | --- |
| Time | Treatment procedure |
| 16:00 pm | *C. camphora* branches were put into Hogland nutrient solution and divided into 5 groups. After adaption for 2 h in a growth chamber at 28^o^C with light intensity of 300 μmol·m^-2^·s^-1^, they were used for further treatments. |
| 18:00 pm | The branches in groups 3-5 were sprayed with 30 μM fosmidomycin (Fos) to block monoterpene synthesis, and each branch was sprayed with 20 ml. The branches in groups 1 and 2 were sprayed with distilled water. After that, all of groups were kept in a growth chamber for 12 h, with the condition of 4 h light (300 μmol·m^-2^·s^-1^, 28^o^C) and 8 h dark (25^o^C). |
| 6:00 am  (the next day) | These groups were put into airtight transparent glass boxes (L×W×H, 35×24×19 cm), with each group in a box. A certain amount of the uppermost monoterpenes was added into groups 4 and 5 to keep the concentration of 1 and 5 µM, respectively, after fully volatilization for 1 h. The uppermost monoterpenes eucalyptol, camphor, linalool and borneol were used to fumigate the corresponding chemotype of *C. camphora*, respectively. |
| 7:00 am  (the next day) | The groups 2-5 were treated with high temperature at 38^o^C, while group 1 was kept at 28^o^C. |
| 12:00 am  (the next day) | At the 5^th^ h during treatment, these groups were immediately changed to new preheating boxes (28^o^C for group 1, and 38^o^C for other groups) for ventilation, and then added into the uppermost monoterpene solution to perform the same treatments (about 25 min for the full volatilization of 5 µM monoterpenes at 38^o^C). |
| 17:00 pm  (the next day) | Investigation of reactive oxygen species (ROS) metabolism, photosynthetic pigment levels, photosynthetic abilities and related gene expression. |

| Supplementary Table 2 Responsive genes in antioxidation in eucalyptol chemotype of *C. camphora* | | | | | | |
| --- | --- | --- | --- | --- | --- | --- |
| Signaling pathway | Gene name | Protein function | FPKM | | | |
|  |  |  | 28^○^C | 38^○^C | Fos+38^○^C | Fos+38^○^C+E5 |
| Antioxidant enzyme | *SOD2* | Encodes **superoxide dismutase** (Fe-Mn family) that exists as a tetramer and scavenges superoxide anion radicals in cells (Zelko et al., 2002). | 0.50±0.24c | 1.12±0.37b | 4.36±1.45a | 0.00±0.00d |
|  | *CAT* | Encodes **catalase** that is an antioxidant enzyme in almost all aerobic organisms. The enzyme translates the H_2_O_2_ to H_2_O and reduce ROS levels (Raza et al., 2021). | 46.08±5.38c | 178.92±8.27b | 229.16±23.27a | 191.27±7.12ab |
| Ascorbate-glutathione cycle | *APX* | Encodes **ascorbate peroxidase** that utilizes ascorbate as specific electron donor to reduce H_2_O_2_ to water. It plays an important role in ROS scavenging in chloroplasts, cytosol, mitochondria and peroxisomes (Caverzan et al., 2012). | 0.28±0.12b | 0.20±0.07b | 2.93±0.98a | 0.00±0.00c |
|  | *gpx* | Encodes **glutathione peroxidase** that is an important enzyme in ascorbate-glutathione cycle for catalyzing the reduction of H_2_O_2_ or organic hydroperoxides to water (Passaia et al., 2014). | 0.22±0.13c | 1.53±0.51b | 3.07±1.02a | 0.00±0.00d |
|  | *GSR* | Encodes **glutathione reductase** that catalyzes glutathione disulfide (GSSG) to glutathione (GSH) depending on NADPH (Couto et al., 2016). | 0.82±0.20c | 4.19±0.38b | 6.41±0.13a | 3.53±0.08b |
| Ascorbate biosynthesis | *GGP* | Encodes **GDP-L-galactose phosphorylase**. It catalyzes the generation of L-galactose-1-P from GDP-L-galactose, the first committed step in L-galactose biosynthesis pathway for L-ascorbate formation (Bulley and Laing, 2016). | 109.51±3.06a | 64.21±2.35b | 61.26±2.30b | 96.86±11.75a |
|  | *GME* | Encodes **GDP-mannose 3',5'-epimerase**. It catalyzes a reversible epimerization of GDP-D-mannose, the committed step in L-ascorbate biosynthesis (Wolucka and Van Montagu, 2003). | 6.31±2.57a | 1.07±0.32b | 0.43±0.14c | 1.36±0.17b |
| Glutathione metabolism | *OPLAH* | Encodes **5-oxoprolinase** (ATP-hydrolyzing). It catalyzes the cleavage of 5-oxo-L-proline to form L-glutamate with coupling hydrolysis of ATP (Almaghlouth et al., 2012). | 15.41±0.60a | 8.73±1.02b | 9.27±0.76b | 17.70±0.85a |
|  | *GST* | Encodes **glutathione S-transferase** that plays an important role in detoxification of toxic substances by conjugating with glutathione (Gullner et al., 2018). | 7.54±1.32c | 89.21±22.19a | 67.65±1.69a | 31.41±2.37b |
|  | *frmA* | Encodes **S-(hydroxymethyl) glutathione dehydrogenase** that belongs to a class III alcohol dehydrogenase and serves function in glutathione-dependent oxidation of formaldehyde (Zhang et al., 2015). | 2.33±0.26b | 3.96±0.51a | 4.52±0.90a | 0.42±0.15c |
| Tocopherol (vitamin E) biosynthesis | *VTE3* | Encodes **MPBQ/MSBQ methyltransferase** that is involved in a key methylation step in tocopherol (vitamin E, VE) and plastoquinone biosynthesis (Mène-Saffrané and DellaPenna, 2010). | 53.10±1.97a | 26.98±2.75c | 22.26±1.58c | 32.36±0.20b |
|  | *E2.1.1.95* | Encodes tocopherol O-methyltransferase that is involved in the synthesis of VE, and methylates gamma- and delta-tocopherol to form beta- and alpha-tocopherol, respectively (Collakova and DellaPenna, 2003). | 47.42±1.32a | 11.57±0.86c | 11.75±0.18c | 15.41±0.48b |
| FPKM: Fragments per kilobase per million mapped reads. 28°C, 38°C, and Fos+38°C: Eucalyptol chemotype of *C. camphora* (EuL) was treated with normal temperature, high temperature, and high temperature with fosmidomycin (Fos) pretreatment, respectively. Fos+38^o^C+C5: EuL pretreated with Fos was fumigated with 5 μM eucalyptol at 38^o^C. Different lowercase letters indicate the significant difference at *P* < 0.05. Means ± SE (n = 3). | | | | | | |

| Supplementary Table 3 Responsive genes in photosynthetic pigment biosynthesis in eucalyptol chemotype of *C. camphora* | | | | | | |
| --- | --- | --- | --- | --- | --- | --- |
| Signaling pathway | Gene name | Protein function | FPKM | | | |
|  |  |  | 28^○^C | 38^○^C | Fos+38^○^C | Fos+38^○^C+E5 |
| Porphyrin and chlorophyll biosynthesis | *chlI* | Encodes a **magnesium chelatase subunit I** that is involved in chlorophyll biosynthesis by catalyzing the insertion of Mg^2+^ into protoporphyrin IX to form Mg-protoporphyrin IX. The magnesium chelatase is a complex of three subunits, including CHLI, CHLD and CHLH (Walker and Weinstein, 1991). | 51.92±4.68a | 20.67±0.63c | 19.88±2.47c | 27.48±1.03b |
|  | *chlD* | Encodes a **magnesium chelatase subunit I** in chlorophyll biosynthesis (Walker and Weinstein, 1991). | 26.28±3.62a | 9.76±0.63c | 9.09±1.89c | 15.21±1.19b |
|  | *EARS* | Encodes **glutamyl-tRNA synthetase**. It catalyzes the synthesis of chlorophyll a from glutamate (Tanaka and Tanaka, 2006). | 7.19±0.58a | 4.68±0.57b | 4.59±0.34b | 6.02±0.32a |
|  | *UROD* | Encodes **uroporphyrinogen decarboxylase**. It is a key enzyme in the formation of chlorophyll and phytochrome, and catalyzes the decarboxylation of urinary porphyrin to form coproporphyrinogen III (Hu et al., 1998). | 8.80±0.11a | 4.71±0.42b | 4.45±0.37b | 7.62±0.96a |
|  | *HCAR* | Encodes **7-hydroxymethyl chlorophyll a reductase**, probable iron-sulfur flavoprotein. It converts 7-hydroxymethyl chlorophyll a to chlorophyll a with ferredoxin as a reducing equivalent (Meguro et al., 2011). | 17.28±1.50a | 8.93±1.53c | 8.58±1.66c | 14.32±0.36b |
|  | *CPOX* | Encodes **coproporphyrinogen III oxidase** that is responsible for the removal of carbon and oxygen atoms from coproporphyrinogen III to form protoporphyrinogen IX (Sun et al., 2011). | 7.09±0.80a | 4.94±0.48b | 5.24±0.05b | 12.26±3.13a |
|  | *acsF* | Encodes **magnesium-protoporphyrin IX monomethyl ester (oxidative) cyclase**. It catalyzes the formation of isocyclic ring in chlorophyll biosynthesis, resulting in the formation of divinylprotochlorophyllide (Pchlide) from magnesium- protoporphyrin IX 13-monomethyl ester (MgPMME) (Bollivar and Beale, 1996). | 218.98±14.77a | 46.29±13.54bc | 44.89±3.76c | 67.96±4.18b |
| Carotenoid biosynthesis | *ZDS* | Encodes **zeta-carotene desaturase** which is a key enzyme in regulating the upstream of β-carotene formation in carotenoid biosynthesis pathway, and catalyzes the dehydrogenation of 9,9'-biscis-ζ-carotene to 7,9,7',9'-tetracis-lycopene (Dong et al., 2007). | 2.83±0.35a | 0.64±0.12bc | 0.33±0.01c | 1.31±0.39b |
|  | *VDE* | Encodes **violaxanthin de-epoxidase** that is a key enzyme in the xanthophyll cycle and catalyzes de-epoxidation of violaxanthin into antheraxanthin and zeaxanthin (Deng et al., 2003). | 24.65±1.87a | 8.42±0.64c | 7.83±1.15c | 15.86±1.57b |
|  | *ZEP* | Encodes zeaxanthin epoxidase that catalyzes the conversion of zeaxanthin into violaxanthin, which is an important part in carotenoid biosynthesis and xanthophyll cycle (Nambara and Marion-Poll, 2005; DellaPenna and Pogson, 2006). | 44.65±1.92a | 20.51±0.34b | 17.41±0.51c | 24.60±1.50b |
| FPKM: Fragments per kilobase per million mapped reads. 28°C, 38°C, and Fos+38°C: Eucalyptol chemotype of *C. camphora* (EuL) was treated with normal temperature, high temperature, and high temperature with fosmidomycin (Fos) pretreatment, respectively. Fos+38^o^C+C5: EuL pretreated with Fos was fumigated with 5 μM eucalyptol at 38^o^C. Different lowercase letters indicate the significant difference at *P* < 0.05. Means ± SE (n = 3). | | | | | | |

| Supplementary Table 4 Responsive genes in photosynthesis in eucalyptol chemotype of *C. camphora* | | | | | | |
| --- | --- | --- | --- | --- | --- | --- |
| Signaling pathway | Gene name | Protein function | FPKM | | | |
|  |  |  | 28^○^C | 38^○^C | Fos+38^○^C | Fos+38^○^C+E5 |
| PSI antenna proteins | *LHCA2* | Encodes **light-harvesting complex I chlorophyll a/b binding protein 2**. The light-harvesting complex I (LHCI) acts as a light receptor, and captures and delivers excitation energy to photosystem I (PSI) (Wientjes et al., 2011). | 1487.83±64.56a | 511.99±58.92c | 447.25±26.97c | 779.30±52.00b |
| PSII antenna proteins | *LHCB2* | Encodes **light-harvesting complex II chlorophyll a/b binding protein 2**. The light-harvesting complex II (LHCI) is also a light receptor, and captures and delivers excitation energy to PSII (Pietrzykowska et al., 2014). | 302.89±55.14a | 86.95±9.64b | 55.02±1.50c | 119.43±18.22b |
|  | *LHCB5* | Encodes **light-harvesting complex II chlorophyll a/b binding protein 5** that is one of the 3 minor highly conserved chlorophyll a/b-binding proteins associated with PSII (Liu et al., 2019). | 203.80±2.73a | 46.71±1.55c | 51.71±7.26c | 70.59±0.46b |
| Oxygen- evolving complex | *psbO* | Encodes **photosystem II** (**PSII) oxygen-evolving enhancer protein 1** that is the largest extrinsic subunit of the membrane-associated photosynthetic redox enzyme in PSII, and is located on the lumen side of thylakoid membranes (Popelkova and Yocum, 2011). | 514.21±6.22a | 135.62±5.08c | 156.98±23.88c | 238.95±15.13b |
|  | *psbP* | Encodes **PSII oxygen-evolving enhancer protein 2**. It is a component of the luminal protein complex for oxygen evolution (Sato, 2010). | 15.23±2.54a | 4.17±0.13c | 3.93±0.86c | 7.73±0.66b |
|  | *psbQ* | Encodes **PSII oxygen-evolving enhancer protein 3** that plays an important role in the luminal oxygen-evolving activity of PSII (Balsera et al., 2003). | 308.22±57.81a | 98.01±10.22bc | 87.47±5.20c | 131.95±11.43b |
| PSII complex | *psbA* | Encodes **PSII P680 reaction center D1 protein** that acts as a primary electron donor of PSII as well as several subsequent electron acceptors (Liere et al., 1995). | 8.83±0.54a | 4.04±0.79bc | 4.46±0.01c | 5.51±0.62b |
|  | *psbK* | Encodes **PSII PsbK protein** that is required for the assembly and stability of PSII complex (Ikeuchi et al., 1991). | 3.54±0.71a | 1.37±0.11b | 1.46±0.24b | 4.6±0.51a |
|  | *psbW* | Encodes **PSII PsbW protein**. It exclusively associated with PSII protein complexes, and plays a role in stabilizing PSII homodimer (García-Cerdán et al., 2011). | 122.40±31.23a | 16.05±5.63c | 10.33±3.62c | 35.45±2.27b |
| PSI complex | *psaA* | Encodes **PSI P700 chlorophyll a apoprotein A1**. PSI converts photonic excitation into a charge separation, and transfers an electron from the donor P700 to the spectroscopically characterized acceptors A0, A1, FX, FA and FB in turn (Fromme et al., 2001). | 0.94±0.09b | 0.53±0.06c | 0.61±0.06c | 13.51±4.90a |
|  | *psaE* | Encodes **PSI subunit IV** that is required for the efficient electron transport between the terminal electron acceptor and ferredoxin. It can stabilize the interaction between PsaC and PSI core, assist the docking of the ferredoxin to PSI and interact with ferredoxin-NADP oxidoreductase (Shimizu et al., 1998). | 347.89±34.67a | 114.81±1.38c | 104.81±13.25c | 205.21±9.88b |
|  | *psaK* | Encodes **PSI subunit X** that is a subunit of PSI. It plays a role in organizing the peripheral light-harvesting complexes on the core antenna of PSI (Varotto et al., 2002). | 76.25±4.67a | 6.85±1.19c | 7.33±2.11c | 13.43±0.47b |
|  | *psaO* | Encodes **PSI subunit PsaO** that is a protein subunit of PSI and plays an important role between the two photosystems (Jensen et al., 2004). | 64.57±4.53a | 25.57±0.46c | 25.42±4.46c | 51.35±4.97b |
| Plastoqui-none (PQ) formation | *HST* | Encodes **homogentisate solanesyltransferase**. It catalyzes the committed step in the biosynthesis of plastoquinone-9 (PQ9) that plays an important role in electron transfer (Hunter et al., 2018). | 17.36±1.08a | 11.58±0.97b | 8.74±0.55c | 11.10±0.43b |
| Cytochrome b_6_-f complex (Cytb_6_-f) | *petA* | Encodes **apocytochrome f**. It is a component in the Cytb_6_-f that mediates electron transfer between PSII and PSI, cyclic electron flow around PSI, and state transitions (Dinkins et al., 1994). | 8.84±0.91b | 4.24±0.45c | 3.84±0.64c | 12.70±0.85a |
|  | *petC* | Encodes **Cytb_6_-f iron-sulfur subunit** that is a component in Cytb_6_-f (Kurisu et al., 2003). | 1265.41±21.86a | 228.24±46.97c | 182.49±13.42c | 400.79±71.33b |
| Plastocyanin | *petE* | Encodes **plastocyanin** that participates in electron transfer between Cytb_6_-f and P700 in PSI (Moore et al., 1991). | 1101.40±8.77a | 305.6±46.89c | 311.51±50.53c | 628.87±39.84b |
| Ferredoxin- NADP^+^ reductase | *petH* | Encodes **ferredoxin--NADP^+^ reductase**. It transfers electrons from ferredoxin (or flavodoxin) to NADP^+^ to generate NADPH (Gómez-Lojero et al., 2003). | 601.50±2.76a | 97.25±2.23c | 78.08±11.84c | 130.28±2.50b |
| ATP synthase | *ATPF1A* | Encodes **F-type H^+^-transporting ATPase subunit α**, a sector of hydrogen-transporting ATP synthase complex (Groth, 2002). | 6.37±1.13a | 3.89±0.57b | 3.54±0.56b | 7.65±0.71a |
|  | *ATPF1B* | Encodes **F-type H^+^-transporting ATPase subunit β**, a sector of hydrogen-transporting ATP synthase complex (Groth, 2002). | 17.32±2.46a | 3.27±0.67c | 2.72±0.38c | 7.53±1.96b |
| Carbon fixation | *MDH1* | Encodes **malate dehydrogenase 1**, a key enzyme in the carbon-fixation pathway of some C4 plants. It catalyzes oxaloacetate to form malic acid in chloroplasts (Gallardo et al., 1995; Musrati et al., 1998). | 8.74±0.20b | 6.03±0.62c | 5.90±0.34c | 13.64±4.55a |
|  | *MDH2* | Encodes **malate dehydrogenase 2** that catalyzes oxaloacetate to form malic acid in chloroplasts in the carbon-fixation pathway of some C4 plants (Gallardo et al., 1995; Musrati et al., 1998). | 45.51±7.45a | 6.40±0.22c | 4.82±0.78d | 13.67±2.90b |
|  | *E1.1.1.82* | Encodes **malate dehydrogenase** (NADP^+^) that converts oxaloacetate into malate in chloroplasts of mesophyll cells for transporting to bundle sheath cells in C4 pathway (Trevanion et al., 1997). | 76.41±1.22a | 22.25±3.43c | 18.42±2.11c | 30.82±1.05b |
|  | *pckA* | Encodes **phosphoenolpyruvate carboxykinase**. It converts oxaloacetate into phosphoenolpyruvate and CO_2_ in C4 plants (Smith and Caruso, 2013). | 0.58±0.03b | 0.13±0.01c | 0.18±0.05c | 2.56±0.72a |
|  | *GAPDH* | Encodes **glyceraldehyde 3-phosphate dehydrogenase**. It catalyzes the reversible reduction and dephosphorylation of 1,3-biphosphoglyceric acid into glyceraldehyde-3-phosphate using NADPH or NADH (Gani et al., 2016). | 9.66±0.18a | 2.24±0.51c | 2.02±0.35c | 4.32±0.09b |
|  | *rbcS* | Encodes **ribulose-bisphosphate carboxylase small chain**. It is a member of the Rubisco small subunit (Evans, 1986). | 8951.66±440.31a | 2764.50±322.28c | 2561.51±513.12c | 4498.87±713.59b |
|  | *ppdK* | Encodes **pyruvate orthophosphate dikinase** that is a cardinal enzyme in C4 pathway, and catalyzes the regeneration of phosphoenolpyruvic acid (PEP), the CO_2_ acceptor (Shi et al., 2020). | 35.74±4.97a | 8.98±0.54c | 4.06±0.40d | 16.85±0.55b |
|  | *TPI* | Encodes **triosephosphate isomerase** that catalyzes the interconversion of the 3-carbon sugars dihydroxyacetone phosphate and D-glyceraldehyde 3-phosphate in the Calvin cycle (Zaffagnini et al., 2014). | 70.16±10.73a | 18.92±2.41bc | 15.40±2.85c | 21.97±1.45b |
|  | *tktA* | Encodes **transketolase**, an essential enzyme in both the Calvin cycle and pentose phosphate pathway (Rocha et al., 2014). | 437.69±7.02a | 240.52±28.44c | 234.82±17.95c | 319.59±0.21b |
|  | *RPE* | Encodes **ribulose-phosphate 3-epimerase** that is required for carbon fixation through the Calvin cycle (Suganami et al., 2018). | 173.78±13.17a | 56.10±6.24c | 49.37±6.15c | 74.29±4.16b |
|  | *rpiA* | Encodes **ribose 5-phosphate isomerase A**. It is an essential enzyme in the Calvin cycle and pentose phosphate pathway, and catalyzes the conversion of ribose 5-phosphate to form ribulose 5-­phosphate (Strange et al., 2009). | 49.87±3.50a | 13.17±1.20c | 8.96±0.62d | 29.81±1.16b |
|  | *PRK* | Encodes **phosphoribulokinase** that is an essential enzyme in photosynthetic eukaryotes. It catalyzes the ATP dependent phosphorylation of ribulose-5-phosphate to form ribulose-1,5-biphosphate, the substrate for ribulose- bisphosphate carboxylase/oxygenase (Rubisco) (Mouche et al., 2002). | 193.44±7.28a | 38.95±5.04c | 30.06±5.59c | 68.34±1.24b |
| FPKM: Fragments per kilobase per million mapped reads. 28°C, 38°C, and Fos+38°C: Eucalyptol chemotype of *C. camphora* (EuL) was treated with normal temperature, high temperature, and high temperature with fosmidomycin (Fos) pretreatment, respectively. Fos+38^o^C+C5: EuL pretreated with Fos was fumigated with 5 μM eucalyptol at 38^o^C. Different lowercase letters indicate the significant difference at *P* < 0.05. Means ± SE (n = 3). | | | | | | |

| Supplementary Table 5 Responsive genes in antioxidation in camphor chemotype of *C. camphora* | | | | | | |
| --- | --- | --- | --- | --- | --- | --- |
| Signaling pathway | Gene name | Protein function | FPKM | | | |
|  |  |  | 28^○^C | 38^○^C | Fos+38^○^C | Fos+38^○^C+C5 |
| Antioxidant enzyme | *SOD1* | Encodes **superoxide dismutase**, Cu-Zn family. SOD1 catalyzes the reaction between superoxide and water to yield oxygen and H_2_O_2_ (Zelko et al., 2002). | 34.91±4.05c | 91.99±6.48b | 127.41±11.83a | 92.38±8.43b |
|  | *CAT* | Encodes **catalase** that is an antioxidant enzyme and exists in almost all aerobic organisms. The enzyme translates the H_2_O_2_ to H_2_O and reduces the reactive oxygen species (ROS) levels (Raza et al., 2021). | 1.71±0.08d | 5.60±0.97b | 11.13±3.71a | 2.95±0.02c |
| Ascorbate-glutathione cycle | *APX* | Encodes **L-ascorbate peroxidase** that utilizes ascorbate as specific electron donor to reduce H_2_O_2_ to water. The importance of the enzyme and ascorbate-glutathione cycle is not restricted to chloroplasts; it also plays a role in ROS scavenging in cytosol, mitochondria and peroxisomes (Caverzan et al., 2012). | 115.63±6.96c | 331.91±36.08b | 568.01±78.41a | 299.29±13.42b |
| Ascorbate biosynthesis | *GGP* | Encodes **GDP-L-galactose phosphorylase**. It catalyzes the generation of L-galactose-1-P from GDP-L-galactose, the first committed step in L-galactose biosynthesis pathway for L-ascorbate formation (Bulley and Laing, 2016). | 19.49±0.65b | 21.24±1.48b | 14.21±0.30c | 35.10±1.59a |
| Glutathione metabolism | *GST* | Encodes **glutathione S-transferase** that is the detoxification of toxic substances by their conjugation with glutathione (Gullner et al., 2018). | 12.68±2.18d | 27.94±2.65b | 120.66±15.98a | 21.75±1.75c |
|  | *frmA* | Encodes **S-(hydroxymethyl) glutathione dehydrogenase** that belongs to a class III alcohol dehydrogenase and functions in the glutathione-dependent oxidation of formaldehyde (Zhang et al., 2015). | 3.04±0.61c | 5.11±0.48b | 6.68±0.57a | 1.00±0.04d |
| Tocopherol (vitamin E) biosynthesis | *VTE3* | Encodes **MPBQ/MSBQ methyltransferase** that is involved in a key methylation step in both VE and plastoquinone synthesis (Mène-Saffrané and DellaPenna, 2010). | 3.67±0.58b | 2.11±0.36c | 1.91±0.11c | 7.58±0.54a |
|  | *E2.1.1.95* | Encodes **tocopherol O-methyltransferase** that is involved in the synthesis of VE. It methylates gamma- and delta-tocopherol to form beta- and alpha-tocopherol, respectively (Collakova and DellaPenna, 2003). | 15.60±2.74b | 11.41±0.90c | 11.53±1.07c | 25.43±1.87a |
| Phenylpropa-noid biosynthesis | *4CL* | Encodes **4-coumarate-CoA ligase** that belongs to the family of ligases, specifically those forming carbon-sulfur bonds as acid-thiol ligases in phenylpropanoid biosynthesis (Allina et al., 1998). | 0.58±0.34a | 0.51±0.15a | 0.00±0.00b | 0.54±0.28a |
|  | *CYP98A* | Encodes **5-O-(4-coumaroyl)-D-quinate 3'-monooxygenase**.  It belongs to the cytochrome P450 family that catalyzes the meta-hydroxylation of p-coumarate derivatives, an important step in the phenylpropanoid pathway (Karamat et al., 2012). | 2.28±0.84a | 0.11±0.07c | 0.00±0.00d | 0.48±0.09b |
| Flavonoid biosynthesis | *FLS* | Encodes **flavonol synthase** that catalyzes the oxidation of dihydroflavonol to form flavonol. The enzyme is the key enzyme responsible for the biosynthesis of flavonols, the most abundant flavonoids (Sun et al., 2019). | 0.50±0.09b | 0.05±0.00c | 0.08±0.04c | 5.08±3.31a |
|  | *HCT* | Encodes **chalcone synthase** that is the key enzyme in the first committed step of the flavonoid biosynthetic pathway and catalyzes the stepwise condensation of 4-coumaroyl-CoA and malonyl-CoA to naringenin chalcone (Deng et al., 2014) | 7.05±0.98a | 3.01±1.35b | 3.04±0.58b | 14.87±7.22a |
| FPKM: Fragments per kilobase per million mapped reads. 28°C, 38°C, and Fos+38°C: Camphor chemotype of *C. camphora* (CmR) was treated with normal temperature, high temperature, and high temperature with fosmidomycin (Fos) pretreatment, respectively. Fos+38^o^C+C5: CmR pretreated with Fos was fumigated with 5 μM camphor at 38^o^C. Different lowercase letters indicate the significant difference at *P* < 0.05. Means ± SE (n = 3). | | | | | | |

| Supplementary Table 6 Responsive genes in photosynthetic pigment biosynthesis in camphor chemotype of *C. camphora* | | | | | | |
| --- | --- | --- | --- | --- | --- | --- |
| Signaling pathway | Gene name | Protein function | FPKM | | | |
|  |  |  | 28^○^C | 38^○^C | Fos+38^○^C | Fos+38^○^C+C5 |
| Porphyrin and chlorophyll biosynthesis | *chlG* | Encodes **chlorophyll a synthase** that catalyzes the production of chlorophyll a. This enzyme belongs to the family of transferases, specifically those transferring aryl or alkyl groups other than methyl groups (Schmid et al., 2002). | 11.02±1.63ab | 10.22±0.46b | 4.69±0.17c | 13.69±0.60a |
|  | *EARS* | *EARS* encodes **glutamyl-tRNA synthetase**. It catalyzes the synthesis of chlorophyll a from glutamate (Tanaka and Tanaka, 2006). | 1.00±0.18a | 0.58±0.28b | 0.08±0.00c | 1.14±0.07a |
|  | *CPOX* | Encodes **coproporphyrinogen III oxidase** that is responsible for removal of carbon and oxygen atoms from coproporphyrinogen III to form protoporphyrinogen IX (Sun et al., 2011). | 0.60±0.04a | 0.25±0.11b | 0.00±0.00c | 0.30±0.16b |
|  | *chlH* | Encodes **magnesium chelatase subunit H** involving in chlorophyll biosynthesis. The enzyme catalyzes the insertion of magnesium ion into protoporphyrin IX to yield Mg-protoporphyrin IX. The magnesium chelatase is a complex of three subunits, including CHLI, CHLD and CHLH (Zhang et al., 2016). | 8.69±1.19b | 5.24±0.26c | 5.55±0.95c | 25.47±4.58a |
|  | *chlI* | Encodes a **magnesium chelatase subunit I** that introduces a magnesium ion into protoporphyrin IX to yield Mg-protorop (Walker and Weinstein, 1991). | 52.45±3.31a | 31.10±4.78b | 28.02±2.92b | 58.75±4.88a |
| Carotenoid biosynthesis | *ispG* | Encodes **(E)-4-hydroxy-3-methylbut-2-enyl-diphosphate synthase** that converts 2C-methyl-D-erythritol 2,4- cyclodiphosphate (ME-2,4cPP) into 1-hydroxy-2-methyl-2- (E)-butenyl 4-diphosphate in methylerythritol-4-phosphate pathway (MEP) for providing precursor for carotenoid formation (Lee et al., 2010). | 7.77±0.41a | 1.24±0.11c | 1.06±0.31c | 2.84±0.55b |
|  | *idi* | Encodes **isopentenyl-diphosphate delta-isomerase** that catalyzes the conversion of isopentenyl diphosphate (IPP) to dimethylallyl diphosphate (DMAPP) in MEP pathway (Pan et al., 2008). | 3.86±0.32a | 2.08±0.26b | 1.40±0.06c | 2.02±0.29b |
|  | *GPS* | Encodes **geranyl diphosphate synthase**, which catalyzes the condensation of DMAPP and IPP to form geranyl diphosphate, the key precursor of monoterpene and carotenoid biosynthesis (Burke et al., 1999). | 0.86±0.10a | 0.58±0.07b | 0.00±0.00c | 0.85±0.23ab |
|  | *crtB* | Encodes **15-cis-phytoene synthase** involving in the biosynthesis of carotenoids. The enzyme catalyzes the condensation of two molecules of geranylgeranyl diphosphate (GGPP) to form prephytoene diphosphate (PPPP) and the subsequent rearrangement of the cyclopropylcarbinyl intermediate to yield the 15-cis-phytoene isomer (Neudert et al., 1998). | 21.51±3.04b | 14.71±0.79c | 14.62±0.87c | 32.15±2.10a |
|  | *ZEP* | Encodes **zeaxanthin epoxidase** that catalyzes the conversion of zeaxanthin into violaxanthin, which is not only an essential part of ABA biosynthesis, but also an important part of carotenoid biosynthesis and the xanthophyll cycle (Nambara and Marion-Poll, 2005; DellaPenna and Pogson, 2006). | 0.21±0.06b | 0.02±0.00c | 0.00±0.00d | 0.90±0.26a |
| FPKM: Fragments per kilobase per million mapped reads. 28°C, 38°C, and Fos+38°C: Camphor chemotype of *C. camphora* (CmR) was treated with normal temperature, high temperature, and high temperature with fosmidomycin (Fos) pretreatment, respectively. Fos+38^o^C+C5: CmR pretreated with Fos was fumigated with 5 μM camphor at 38^o^C. Different lowercase letters indicate the significant difference at *P* < 0.05. Means ± SE (n = 3). | | | | | | |

| Supplementary Table 7 Responsive genes in photosynthesis in camphor chemotype of *C. camphora* | | | | | | |
| --- | --- | --- | --- | --- | --- | --- |
| Signaling pathway | Gene name | Protein function | FPKM | | | |
|  |  |  | 28^○^C | 38^○^C | Fos+38^○^C | Fos+38^○^C+C5 |
| PSI antenna proteins | *LHCA1* | Encodes **light-harvesting complex I chlorophyll a/b binding protein 1**. The light-harvesting complex (LHC) functions as a light receptor, and it captures and delivers excitation energy to photosystems with which it is closely associated (Liu et al., 2013). | 217.13±23.45a | 169.18±24.44ab | 94.35±18.42c | 158.51±18.80b |
|  | *LHCA2* | Encodes **light-harvesting complex I chlorophyll a/b binding protein 2**. LHC functions as a light receptor, and captures and delivers excitation energy to PSI with which it is closely associated (Wientjes et al., 2011). | 29.55±8.53ab | 21.38±0.50b | 8.83±1.02c | 32.39±0.82a |
| PSII antenna proteins | *LHCB1* | Encodes **light-harvesting complex II chlorophyll a/b binding protein 1**. Most of the photons that are converted into biochemical energy and biomass through photosynthesis are harvested by the major light-harvesting chlorophyll a/b binding antenna complex light-harvesting complex II (LHCII), which is one of the most abundant proteins on earth (Liu et al., 2013). | 251.00±36.59a | 142.43±32.87b | 56.91±6.88c | 117.84±11.52b |
|  | *LHCB2* | Encodes **light-harvesting complex II chlorophyll a/b binding protein 2**. Most of the photons that are converted to biochemical energy and biomass through photosynthesis are harvested by the major light-harvesting chlorophyll a/b binding antenna complex LHCII (Pietrzykowska et al., 2014). | 12.38±4.13a | 0.15±0.02c | 0.06±0.00d | 1.43±0.75b |
| Oxygen-  evolving complex | *psbP* | Encodes **PSII oxygen-evolving enhancer protein 2**. It is a component of the luminal protein complex associated with PSII. PsbP is a thylakoid lumen protein and is involved in oxygen evolution in PSII (Sato, 2010). | 4213.98±719.00a | 1897.35±59.19c | 1517.35±34.79d | 2638.22±205.77b |
|  | *psbQ* | Encodes **PSII oxygen-evolving enhancer protein 3** that plays an important role in the lumenal oxygen-evolving activity of PSII in higher plants and green algae (Balsera et al., 2003). | 191.36±9.82a | 95.18±10.03b | 80.02±8.28c | 111.00±8.81b |
| PSII complex | *psbK* | Encodes **photosystem II (PSII) PsbK protein** that is required for the stable assembly and/or stability of the PSII complex (Ikeuchi et al., 1991). | 1033.61±19.75a | 573.91±38.81b | 434.52±13.37c | 951.77±71.00a |
|  | *psbS* | Encodes **PSII 22kDa protein**. The PsbS protein functions in the regulation of photosynthetic light harvesting in PSII (Niyogi et al., 2005). | 628.34±147.94a | 253.47±25.51b | 180.86±23.93c | 615.42±88.08a |
|  | *psbW* | Encodes **PSII PsbW protein**. It exclusively associated with PSII protein complexes, and plays a role in the stabilization of the PSII homodimer (García-Cerdán et al., 2011). | 49.29±7.75a | 28.60±3.57b | 15.88±2.07c | 43.13±4.60a |
| PSI complex | *psaB* | Encodes **PSI P700 chlorophyll a apoprotein A2**. The function of this protein is the same with PSI P700 chlorophyll a apoprotein A1 (Fromme et al., 2001). | 2.74±0.01a | 2.69±0.10a | 1.48±0.24b | 3.16±0.37a |
| Ferredoxin-  NADP^+^ reductase | *petH* | Encodes **ferredoxin--NADP^+^ reductase**. It transfers electrons from ferredoxin (or flavodoxin) to NADP^+^ to generate NADPH (Gómez-Lojero et al., 2003). | 301.33±51.70a | 138.13±12.89c | 125.89±5.66c | 218.98±21.71b |
| ATP synthase | *ATPF1B* | Encodes **F-type H^+^-transporting ATPase subunit β**, a sector of hydrogen-transporting ATP synthase complex (Groth, 2002). | 31.04±7.74a | 9.54±0.40c | 4.19±0.52d | 18.42±1.52b |
|  | *ATPF1G* | Encodes **F-type H^+^-transporting ATPase subunit gamma**, a sector of hydrogen-transporting ATP synthase complex (Groth, 2002). | 16.55±2.80a | 1.67±0.56b | 0.00±0.00c | 2.77±1.22b |
| Carbon fixation | *ppc* | Encodes **phosphoenolpyruvate carboxylase**, the key enzyme in C4 metabolism. It catalyzes the irreversible β-carboxylation of phosphoenolpyruvate (PEP) in a broad range of metabolic schemes (Heldt et al., 2011). | 0.98±0.33a | 0.00±0.00b | 0.00±0.00b | 0.86±0.14a |
|  | *MDH2* | Encodes **malate dehydrogenase 2** that catalyzes oxaloacetate to form malic acid in chloroplasts in the carbon-fixation pathway of some C4 plants (Musrati et al., 1998). | 7.65±3.77a | 0.23±0.03c | 0.00±0.00d | 1.01±0.31b |
|  | *GOT2* | Encodes **aspartate aminotransferase 2** that relates with nitrogen metabolism and C4-pathway photosynthesis (Funakoshi et al., 2008). | 1.84±0.87a | 0.26±0.13b | 0.00±0.00c | 1.12±0.51a |
|  | *maeB* | Code for **malate dehydrogenase (oxaloacetate- decarboxylating) depending on NADP^+^**. It is one of three decarboxylation enzymes used in the inorganic carbon concentrating mechanisms of C4 and CAM plants. Malate is oxidized to pyruvate and CO_2_, and NADP^+^ is reduced to NADPH (Detarsio et al., 2003). | 1.77±0.37a | 0.31±0.07b | 0.00±0.00c | 0.51±0.23b |
|  | *pckA* | Encodes **phosphoenolpyruvate carboxykinase** depending on ATP. It converts oxaloacetate into phosphoenolpyruvate and CO_2_ in C4 plants (Smith and Caruso, 2013). | 14.15±4.13a | 0.20±0.03c | 0.00±0.00d | 1.67±0.81b |
|  | *GAPDH* | Encodes **glyceraldehyde 3-phosphate dehydrogenase** (phosphorylating) that catalyzes the reversible reduction and dephosphorylation of 1,3-biphosphoglyceric acid into glyceraldehyde-3-phosphate (Gani et al., 2016). | 9.81±4.36a | 0.32±0.11c | 0.09±0.02d | 1.00±0.44b |
|  | *rbcS* | Encodes **ribulose-bisphosphate carboxylase small chain**. Ribulose-bisphosphate carboxylase/oxygenase (Rubisco) is the primary enzyme responsible for autotrophy and is a bi-functional enzyme catalyzing both the carboxylation of D-ribulose-1,5-bisphosphate that initiates photosynthetic CO_2_ fixation and the oxygenation of RuBP that starts the nonessential photorespiratory pathway. Its holoenzyme in green algae and higher plants consists of eight large subunits (LSUs) encoded by the chloroplast gene *rbcL* and eight small subunits (SSUs) encoded by the nuclear gene *rbcS* (Stec, 2012). | 5514.76±929.91b | 2416.2±442.39c | 746.35±76.03d | 7324.27±489.35a |
|  | *rbcL* | Encodes **ribulose-bisphosphate carboxylase large chain**. It is a member of Rubisco large subunit (Stec, 2012). | 10.60±0.66a | 8.01±1.51b | 4.19±0.37c | 9.58±0.86ab |
|  | *TPI* | Encodes **triosephosphate isomerase** that catalyzes the interconversion of the three-carbon sugars dihydroxyacetone phosphate (DHAP) and D-glyceraldehyde 3-phosphate (GAP) in Calvin cycle and glucolysis (Zaffagnini et al., 2014). | 3.85±1.51a | 0.42±0.14c | 0.19±0.01d | 1.13±0.38b |
|  | *tktA* | Encodes **transketolase**, an essential enzyme of both the Calvin cycle and pentose phosphate pathway (Rocha et al., 2014). | 2.58±1.05a | 0.57±0.23b | 0.00±0.00c | 1.64±0.85ab |
|  | *rpiA* | Encodes **ribose 5-phosphate isomerase A**. It is an essential enzyme in the Calvin cycle and pentose phosphate pathway. Specifically, the enzyme catalyzes the reversible conversion of ribose 5-phosphate to ribulose 5-­phosphate (Strange et al., 2009). | 54.83±13.29a | 16.49±1.10b | 15.81±7.92b | 37.57±5.83a |
|  | *PRK* | Encodes **phosphoribulokinase** that is an essential enzyme of photosynthetic eukaryotes. It is active in the plastid-located Calvin cycle and catalyzes the ATP-dependent phosphorylation of ribulose-5-phosphate to form ribulose-1,5-biphosphate (Mouche et al., 2002). | 33.58±0.90a | 9.86±1.15c | 10.17±0.80c | 19.98±2.13b |
| FPKM: Fragments per kilobase per million mapped reads. 28°C, 38°C, and Fos+38°C: Camphor chemotype of *C. camphora* (CmR) was treated with normal temperature, high temperature, and high temperature with fosmidomycin (Fos) pretreatment, respectively. Fos+38^o^C+C5: CmR pretreated with Fos was fumigated with 5 μM camphor at 38^o^C. Different lowercase letters indicate the significant difference at *P* < 0.05. Means ± SE (n = 3). | | | | | | |

**References for Supplementary Tables 2-7**

Allina, S. M., Pri-Hadash, A., Theilmann, D. A., Ellis, B. E., Douglas, C. J., 1998. 4-Coumarate: coenzyme A ligase in hybrid poplar: Properties of native enzymes, cDNA cloning, and analysis of recombinant enzymes. Plant Physiol. 116: 743–754.

Almaghlouth, I.A., Mohamed, J.Y., Al-Amoudi, M., Al-Ahaidib, L., Al-Odaib, A., Alkuraya, F.S., 2012. 5-Oxoprolinase deficiency: report of the first human *OPLAH* mutation. Clin. Genet. 82, 193–196.

Balsera, M., Arellano, J.B., Gutierrez, J.R., Heredia, P., Revuelta, J.L., Javier, D.L.R., 2003. Structural analysis of the PsbQ protein of photosystem II by Fourier transform infrared and circular dichroic spectroscopy and by bioinformatic methods. Biochemistry 42, 1000–1007.

Bollivar, D.W., Beale, S.I., 1996. The chlorophyll biosynthetic enzyme Mg-protoporphyrin IX monomethyl ester (oxidative) cyclase (Characterization and partial purification from *Chlamydomonas reinhardtii* and *Synechocystis* sp. PCC 6803). Plant Physiol. 112, 105–114.

Bulley, S., Laing, W., 2016. The regulation of ascorbate biosynthesis. Curr. Opin. Plant Biol. 33, 15–22.

Burke, C. C., Wildung, M. R., Croteau, R., 1999. Geranyl diphosphate synthase: cloning, expression, and characterization of this prenyltransferase as a heterodimer. Proc. Natl. Acad. Sci. USA 96: 13062-13067.

Caverzan, A., Passaia, G., Rosa, S.B., Ribeiro, C.W., Lazzarotto, F., Margis-Pinheiro, M., 2012. Plant responses to stresses: role of ascorbate peroxidase in the antioxidant protection. Genet Mol. Biol. 35, 1011–1019.

Collakova, E., DellaPenna, D., 2003. Homogentisate phytyltransferase activity is limiting for tocopherol biosynthesis in *Arabidopsis*. Plant Physiol. 131, 632–642.

Couto, N., Wood, J., Barber, J., 2016. The role of glutathione reductase and related enzymes on cellular redox homoeostasis network. Free Radical Bio. Med. 95, 27–42.

DellaPenna, D., Pogson, B.J. 2006. Vitamin synthesis in plants: tocopherols and carotenoids. Annu. Rev. Plant Biol. 57, 711–738.

Deng, X., Bashandy, H., Ainasoja, M., Kontturi, J., Pietiäinen, M., Laitinen, R. A. E., Albert, V. A., Valkonen, J. P. T., Elomaa, P., Teeri, T. H. 2014. Functional diversification of duplicated chalcone synthase genes in anthocyanin biosynthesis of *Gerbera hybrida*. New Phytol. 201: 1469-1483.

Deng, Y., Lin, R, Jing, Y., Wang, Q., Li, L., Liu, B., Kuang, T., 2003. Expression of *VDE* gene integrated into tobacco genome in antisense and overexpressed ways. Photosynthetica 41, 137–141.

Detarsio, E., Wheeler, M. C., Bermúdez, V. A. C., Andreo, C. S., Drincovich, M. F., 2003. Maize C4 NADP-malic enzyme expression in *Escherichia coli* and characterization of site-directed mutants at the putative nucleoside-binding sites. J. Biol. Chem. 278: 13757–13764.

Dinkins, R.D., Bandaranayake, H., Green, B.R., Griffiths, A.J.F., 1994. A nuclear photosynthetic electron transport mutant of *Arabidopsis thaliana* with altered expression of the chloroplast *petA* gene. Curr. Genet. 25, 282–288.

Dong, H., Deng, Y., Mu, J., Lu, Q., Wang, Y., Xu, Y., Chu, C., Chong, K., Lu, C., Zuo, J., 2007. The *Arabidopsis* spontaneous cell death1 gene, encoding a zeta-carotene desaturase essential for carotenoid biosynthesis, is involved in chloroplast development, photoprotection and retrograde signaling. Cell Res. 5, 458–470.

Evans, J.R., 1986. The relationship between carbon-dioxide-limited photosynthetic rate and ribulose-1,5- bisphosphate-carboxylase content in two nuclear-cytoplasm substitution lines of wheat, and the coordination of ribulose-bisphosphate-carboxylation and electron-transport c. Planta 167, 351–358.

Fromme, P., Jordan, P., Krauß, N., 2001. Structure of photosystem I. BBA-Bioenergetics 1507, 5–31.

Funakoshi, M., Sekine, M., Katane, M., Furuchi, T., Yohda, M., Yoshikawa, T., Homma, H., 2008. Cloning and functional characterization of *Arabidopsis thaliana* D-amino acid aminotransferase--D-aspartate behavior during germination. FEBS J. 275: 1188–1200.

Gallardo, F., Miginiac-Maslow, M., Sangwan, R.S., Decottignies, P., Keryer, E., Dubois, F., Bismuth, E., Galvez, S., Sangwan-Norreel, B., Gadal, P., Crétin, C., 1995. Monocotyledonous C4 NADP^+^-malate dehydrogenase is efficiently synthesized, targeted to chloroplasts and processed to an active form in transgenic plants of the C3 dicotyledon tobacco. Planta 197, 324–332.

Gani, Z., Boradia, V.M., Ram, J.R., Suryavanshi, P.M., Patil, P., Kumar, S., Singh, R., Raje, M., Raje, C.L., 2016. Purification and characterization of glyceraldehyde-3-phosphate-dehydrogenase (GAPDH) from pea seeds. Protein Expr. Purif. 127, 22–27.

García-Cerdán, J.G., Kovács, L., Tóth, T., Kereïche, S., Aseeva, E., Boekema, E.J., Mamedov, F., Funk, C., Schröder, W.P., 2011. The PsbW protein stabilizes the supramolecular organization of photosystem II in higher plants. Plant J. 65, 368–381.

Gómez-Lojero, C., Pérez-Gómez, B., Shen, G., Schluchter, W.M.., Bryant, D.A., 2003. Interaction of ferredoxin: NADP^+^ oxidoreductase with phycobilisomes and phycobilisome substructures of the cyanobacterium *Synechococcus* sp. strain PCC 7002. Biochemistry 42, 13800–13811.

Groth, G., 2002. Structure of spinach chloroplast F1-ATPase complexed with the phytopathogenic inhibitor tentoxin. Proc. Natl. Acad. Sci. USA 99, 3464–3468.

Gullner, G., Komives, T., Király, L., Schröder, P., 2018. Glutathione S-transferase enzymes in plant-pathogen interactions. Front. Plant Sci. 9, 1836.

Heldt, H.-W., Piechulla, B., 2011. Photosynthesis implies the consumption of water. In: Heldt H-W, Piechulla B (eds.), Plant Biochemistry (Fourth Edition). Academic Press, San Diego, pp. 211–239.

Hu, G., Yalpani, N., Briggs, S.P., Johal, G.S., 1998. A porphyrin pathway impairment is responsible for the phenotype of a dominant disease lesion mimic mutant of maize. Plant Cell 10, 1095–1105.

Hunter, C.T., Saunders, J.W., Magallanes-Lundback, M., Christensen, S.A., Willett, D., Stinard, P.S., Li, Q.B., Lee, K., DellaPenna, D., Koch, K.E., 2018. Maize w3 disrupts homogentisate solanesyl transferase (ZmHst) and reveals a plastoquinone-9 independent path for phytoene desaturation and tocopherol accumulation in kernels. Plant J. 93, 799–813.

 Ikeuchi, M., Eggers, B., Shen, G.Z., Webber, A., Yu, J.J., Hirano, A., Inoue, Y., Vermaas, W., 1991. Cloning of the *psbK* gene from *Synechocystis* sp. PCC 6803 and characterization of photosystem ii in mutants lacking PSII-K. J. Biol. Chem. 266, 11111–11115.

Jensen, P.E., Haldrup, A., Zhang, S., Scheller, H.V., 2004. The PSI-O subunit of plant photosystem I is involved in balancing the excitation pressure between the two photosystems. J. Biol. Chem. 279, 24212–24217.

Karamat, F., Olry, A., Doerper, S., Vialart, G., Ullmann, P., Werck-Reichhart, D., Bourgaud, F., Hehn, A., 2012. CYP98A22, a phenolic ester 3'-hydroxylase specialized in the synthesis of chlorogenic acid, as a new tool for enhancing the furanocoumarin concentration in *Ruta graveolens*. BMC Plant Biol. 12: 152.

Kurisu, G., Zhang, H., Smith, J.L., Cramer, W.A., 2003. Structure of the cytochrome b_6_f complex of oxygenic photosynthesis: tuning the cavity. Science 302, 1009–1014.

Lee, M., Grwert, T., Quitterer, F., Rohdich, F., Rohdich, F., Eppinger, J., Eisenreich, W., Bacher, A., Groll, M., 2010. Biosynthesis of isoprenoids: crystal structure of the [4Fe-4S] cluster protein IspG. J. Mol. Biol. 404: 600-610.

Liere, K., Kestermann, M., Müller, U., Link, G., 1995. Identification and characterization of the *Arabidopsis thaliana* chloroplast DNA region containing the genes *psbA*, *trnH* and *rps19'.* Curr. Genet. 28, 128–130.

Liu, M., Zhang, S., Hu, J., Sun, W., Padilla, J., He, Y., Li, Y., Yin, Z., Liu, X., Wang, W., Shen, D., Li, D., Zhang, H., Zheng, X., Cui, Z., Wang, G.L., Wang, P., Zhou, B., Zhang, Z., 2019. Phosphorylation-guarded light-harvesting complex II contributes to broad-spectrum blast resistance in rice. Proc. Natl. Acad. Sci. USA 116, 17572–17577.

Liu, R., Xu, Y., Jiang, S., Lu, K., Lu, Y., Feng, X., Wu, Z., Liang, S., Yu, Y., Wang, X., Zhang, D., 2013. Light-harvesting chlorophyll a/b-binding proteins, positively involved in abscisic acid signaling, require a transcription repressor, WRKY40, to balance their function. J. Exp. Bot. 64: 5443–5456.

Meguro, M., Ito, H., Takabayashi, A., Tanaka, R., Tanaka, A., 2011. Identification of the 7-hydroxymethyl chlorophyll a reductase of the chlorophyll cycle in *Arabidopsis*. Plant Cell 23, 3442–3453.

Mène-Saffrané, L., DellaPenna, D., 2010. Biosynthesis, regulation and functions of tocochromanols in plants. Plant Physiol. Biochem. 48, 301–309.

Moore, J.M., Lepre, C.A., Gippert, G.P., Chazin, W.J., Case, D.A., Wright, P.E., 1991. High-resolution solution structure of reduced French bean plastocyanin and comparison with the crystal structure of poplar plastocyanin. J. Mol. Biol. 221, 533–555.

 Mouche, F., Gontero, B., Callebaut, I., Mornon, J.P., Boisset, N., 2002. Striking conformational change suspected within the phosphoribulokinase dimer induced by interaction with *GAPDH*. J. Biol. Chem. 277, 6743–6749.

Musrati, R.A., Kollárová, M., Mernik, N., Mikulásová, D., 1998. Malate dehydrogenase: distribution, function and properties. Gen. Physiol. Biophys. 17, 193–210.

Nambara, E., Marion-Poll, A. 2005. Abscisic acid biosynthesis and catabolism. Annu. Rev. Plant Biol. 56, 165–185.

Neudert, U., Martinez-Ferez, I. M., Fraser, P. D., Sandmann, G., 1998. Expression of an active phytoene synthase from *Erwinia uredovora* and biochemical properties of the enzyme. BBA - Lipid Lipid Met. 1392: 51–58.

Niyogi, K. K., Li, X. P., Rosenberg, V., Jung, H. S., 2005. Is PsbS the site of non-photochemical quenching in photosynthesis? J. Exp. Bot. 56: 375-382.

Pan, X., Chen, M., Liu, Y., Wang, Q., Zeng, L., Li, L., Liao, Z., 2008. A new isopentenyl diphosphate isomerase gene from *Camptotheca acuminata*: cloning, characterization and functional expression in *Escherichia coli*. DNA Sequence 19: 98-105.

Passaia, G., Caverzan, A., Fonini, L.S., Carvalho, F.E.L., Silveira, J.A.G., Margis-Pinheiro, M., 2014. Chloroplastic and mitochondrial *gpx* genes play a critical role in rice development. Biol. Plantarum. 58, 375–378.

Pietrzykowska, M., Suorsa, M., Semchonok, D.A., Tikkanen, M., Boekema, E.J., Aro, E.M., Janssona, S., 2014. The light-harvesting chlorophyll a/b binding proteins Lhcb1 and Lhcb2 play complementary roles during state transitions in *Arabidopsis*. Plant Cell 26, 3646–3660.

Raza, A., Su, W., Gao, A., Mehmood, S.S., Hussain, M.A., Nie, W., Lv, Y., Zou, X., Zhang, X., 2021. Catalase (*CAT*) gene family in rapeseed (*Brassica napus* L.): genome-wide analysis, identification, and expression pattern in response to multiple hormones and abiotic stress conditions. Int. J. Mol. Sci. 22, 4281.

Rocha, A.G., Mehlmer, N., Stael, S., Mair, A., Parvin, N., Chigri, F., Teige, M., Vothknecht, U.C., 2014. Phosphorylation of *Arabidopsis* transketolase at Ser428 provides a potential paradigm for the metabolic control of chloroplast carbon metabolism. Biochem. J. 458, 313–322.

Popelkova, H., Yocum, C.F., 2011. *PsbO*, the manganese-stabilizing protein: analysis of the structure–function relations that provide insights into its role in photosystem II. J. Photoch. Photobio. B. 104, 179–190.

Sato, N., 2010. Phylogenomic and structural modeling analyses of the *PsbP* superfamily reveal multiple small segment additions in the evolution of photosystem II-associated PsbP protein in green plants. Mol. Phylogenet. Evol. 56, 176–186.

Schmid, H. C., Rassadina, V., Oster, U., Schoch, S., Rudiger, W. 2002. Pre-loading of chlorophyll synthase with tetraprenyl diphosphate is an obligatory step in chlorophyll biosynthesis. Biol. Chem. 383: 1769–1778.

Shimizu, T., Mitsue, M.T., Yamamoto, N., Ohashi, Y., Kobayashi, S., 1998. Construction of antisense transgenic plants of photosystem I *psaD* and *psaE*. Plant Cell Physiol. 39, S11–S11.

Shi, W., Yue, L., Guo, J., Wang, J., Yuan, X., Dong, S., Guo, J., Guo, P., 2020. Identification and evolution of C4 photosynthetic pathway genes in plants. BMC Plant Biol. 20, 132.

Smith, A.A., Caruso, A., 2013. In silico characterization and homology modeling of a cyanobacterial phosphoenolpyruvate carboxykinase enzyme. Struct. Biol. 2013, 1–10.

Stec, B., 2012. Structural mechanism of RuBisCO activation by carbamylation of the active site lysine. Proc. Natl. Acad. Sci. USA 109: 18785-18790.

Strange, R.W., Antonyuk, S.V., Ellis, M.J., Bessho, Y., Kuramitsu, S., Yokoyama, S., Hasnain, S.S., 2009. The structure of an archaeal ribose-5-phosphate isomerase from *Methanocaldococcus jannaschii* (MJ1603). Acta Crystallogr. F. 65, 1214–1217.

Suganami, M., Suzuki, Y., Sato, T., Makino, A., 2018. Relationship between rubisco activase and rubisco contents in transgenic rice plants with overproduced or decreased rubisco content. Soil Sci. Plant Nutr. 64, 352–359.

Sun, C., Liu, L., Tang, J., Lin, A., Zhang, F., Fang, J., Zhang, G., Chu. C., 2011. *RLIN1*, encoding a putative coproporphyrinogen III oxidase, is involved in lesion initiation in rice*.* J. Genet. Genomics 38, 29–37.

Sun, Y. J., He, J. M., Kong, J. Q. 2019. Characterization of two flavonol synthases with iron-independent flavanone 3-hydroxylase activity from *Ornithogalum caudatum* Jacq. BMC Plant Biol. 19: 195.

Tanaka, A., Tanaka, R., 2006. Chlorophyll metabolism. Curr. Opin. Plant Biol. 9, 248–255.

Trevanion, S.J., Furbank, R.T., Ashton, A.R., 1997. NADP-malate dehydrogenase in the C4 plant *Flaveria bidentis* (Cosense suppression of activity in mesophyll and bundle-sheath cells and consequences for photosynthesis). Plant Physiol. 113, 1153–1165.

Varotto, C., Pesaresi, P., Jahns, P., Leßnick, A., Tizzano, M., Schiavon, F., Salamini, F., Leister, D., 2002. Single and double knockouts of the genes for photosystem I subunits g, k, and h of *Arabidopsis*. Effects on photosystem I composition, photosynthetic electron flow, and state transitions. Plant Physiol. 129, 616–624.

Walker, C.J., Weinstein, J.D., 1991. *In vitro* assay of the chlorophyll biosynthetic enzyme Mg-chelatase: resolution of the activity into soluble and membrane-bound fractions. Proc. Natl. Acad. Sci. USA 88, 5789–5793.

Wientjes, E., van Stokkum, I.M., van Amerongen, H., Croce, R., 2011. The role of the individual Lhcas in photosystem I excitation energy trapping. Biophys. J. 101, 745–754.

Wolucka, B.A., Van Montagu, M., 2003. GDP-mannose 3′,5′-epimerase forms GDP-l-gulose, a putative intermediate for the *de novo* biosynthesis of vitamin C in plants.  J. Biol. Chem. 278, 47483–47490.

Zaffagnini, M., Michelet, L., Sciabolini, C., Di Giacinto, N., Morisse, S., Marchand, C.H., Trost, P., Fermani, S., Lemaire, S.D., 2014. High-resolution crystal structure and redox properties of chloroplastic triosephosphate isomerase from *Chlamydomonas reinhardtii*. Mol. Plant 7, 101–120.

Zelko, I.N., Mariani, T.J., Folz, R.J., 2002. Superoxide dismutase multigene family: a comparison of the CuZn-SOD (*SOD1*), Mn-SOD (*SOD2*), and EC-SOD (*SOD3*) gene structures, evolution, and expression. Free Radic. Biol. Med. 33, 337–349.

Zhang, Z., Wang, J., Chai, R., Qiu, H., Jiang, H., Mao, X., Wang, Y., Liu, F., Sun, G., 2015. An S-(Hydroxymethyl) glutathione dehydrogenase is involved in conidiation and full virulence in the rice blast fungus *Magnaporthe oryzae*. PloS One 10, e0120627.

Zhang, Z. W., Wu, Z. L., Feng, L. Y., Dong, L. H., Song, A. J., Yuan, M., Chen, Y. E., Zeng, J., Chen, G. D., Yuan, S. 2016. Mg-protoporphyrin IX signals enhance plant's tolerance to cold stress. Front. Plant Sci. 7: 1545.
